# KDOSS-net: Knowledge distillation-based outpainting and semantic segmentation network for crop and weed images

**DOI:** 10.1016/j.plaphe.2025.100098

**Published:** 2025-08-20

**Authors:** Sang Hyo Cheong, Sung Jae Lee, Su Jin Im, Juwon Seo, Kang Ryoung Park

**Affiliations:** Division of Electronics and Electrical Engineering, Dongguk University, 30 Pildong-ro 1-gil, Jung-gu, Seoul, 04620, Republic of Korea

**Keywords:** Crops and weeds, Limited field of view, Object prediction-guided image outpainting and semantic segmentation network, Knowledge distillation, Pesticide recommendation

## Abstract

Weed management plays a crucial role in increasing crop yields. Semantic segmentation, which classifies each pixel in an image captured by a camera into categories such as crops, weeds, and background, is a widely used method in this context. However, conventional semantic segmentation methods rely solely on pixel information within the camera's field of view (FOV), hindering their ability to detect weeds outside the visible area. This limitation can lead to incomplete weed removal and inefficient herbicide application. Incorporating information beyond the FOV in crop and weed segmentation is therefore essential for effective herbicide usage. Nevertheless, existing research on crop and weed segmentation has largely overlooked this limitation. To address this issue, we propose the knowledge distillation–based outpainting and semantic segmentation network (KDOSS-Net) for crop and weed images, a novel framework that enhances segmentation accuracy by leveraging information beyond the FOV. KDOSS-Net consists of two parts: the object prediction–guided outpainting and semantic segmentation network (OPOSS-Net), which serves as the teacher model by restoring areas outside the FOV and performing semantic segmentation, and the semantic segmentation without outpainting network (SSWO-Net), which serves as the student model, directly performing segmentation without outpainting. Through knowledge distillation (KD), the student model learns from the teacher's outputs, which results in a lightweight yet highly accurate segmentation network that is suitable for deployment on agricultural robots with limited computing power. Experiments on three public datasets—Rice seedling and weed, CWFID, and BoniRob—yielded mean intersection over union (*mIOU*) scores of 0.6315, 0.7101, and 0.7524, respectively. These results demonstrate that KDOSS-Net achieves higher accuracy than existing state-of-the-art (SOTA) segmentation models while significantly reducing computational overhead. Furthermore, the weed information extracted using our method is automatically linked as input to the open-source large language and vision assistant (LLaVA), enabling the development of a system that recommends optimal herbicide strategies tailored to the detected weed class.

## 1 Introduction

In agriculture, weeds represent one of the primary challenges that hinder crop growth and reduce harvesting efficiency. Various weed management techniques, including herbicide application, have been developed to address this issue. For effective weed control that minimizes harm to crops, precise detection and classification of weeds are essential. However, crops and weeds often exhibit similar shapes and colors, making it difficult to distinguish between them visually. Therefore, accurate identification requires sophisticated approaches. Recently, advances in deep learning–based semantic segmentation techniques [1] have led to their active application in plant phenotyping research [2]. Semantic segmentation enables pixel-level classification, allowing for the precise localization of crops and weeds in images captured by cameras—a critical capability for targeted herbicide application in agricultural settings. Conventional segmentation methods, however, rely exclusively on the information available within the camera's field of view (FOV). In real-world agricultural environments, this inherent limitation makes it difficult to detect all existing weeds. Herbicide applications then become confined to the weeds detected within the FOV, leaving those outside untreated and potentially undermining the overall weed control effort. Moreover, if herbicides are applied only to weeds visible within the FOV, undetected weeds beyond that area may persist and spread. This persistence may necessitate additional weed management interventions and reduce the overall efficiency of herbicide usage. In cases where undetected weeds encroach upon crop boundaries, there is a risk of reduced overall yield. Fig. S1 (in the Supplementary Materials) visually compares the semantic segmentation results of a limited FOV image with those of a full original image using a U-Net model [3] trained on original images. The figure clearly shows that weeds located outside the FOV—particularly in the upper region of the limited FOV image—are not recognized, resulting in inefficient herbicide spraying. To ensure more effective crop and weed segmentation in agricultural environments, it is necessary to incorporate information from regions beyond the FOV into the segmentation process. One approach is to use image outpainting to reconstruct areas beyond the current FOV and then perform semantic segmentation on the extended image. However, existing outpainting methods face a significant challenge: the computational cost increases considerably when predicting complex objects, making real-time deployment on farming robots impractical [4,30]. To address the trade-off between segmentation accuracy and computational efficiency, we propose the knowledge distillation–based outpainting and semantic segmentation network (KDOSS-Net) for crops and weeds. Our method employs a teacher–student architecture, where the teacher model—a complex, high-capacity network—performs outpainting and learns rich semantic information, while the student model performs segmentation directly without outpainting. Through knowledge distillation, the student model inherits the teacher's knowledge, thereby achieving high segmentation accuracy with significantly lower computational requirements. In this paper, we categorize previous crop and weed segmentation studies into two groups. The first group comprises segmentation techniques that do not consider the limited FOV, and the second includes those that incorporate FOV limitations. These categories are described in detail in the following subsections.

### 1.1 Segmentation not considering the limited FOV

Previous research on crop and weed segmentation has primarily relied on detecting weeds using only the information within the camera's FOV. These studies can be broadly divided into handcrafted feature–based methods and deep feature–based methods.

#### 1.1.1 Handcrafted feature-based methods

Handcrafted feature–based methods classify crops and weeds by leveraging manually designed features such as color, shape, and texture. For example, one study [6] converted the RGB color space to the hue, saturation, and intensity (HSI) space to analyze color differences and then applied classification based on Mahalanobis distance, which achieved robust performance under varying lighting conditions. Another study [7] utilized chlorophyll reflectance from near-infrared (NIR) images to generate normalized difference vegetation index (NDVI) images for threshold-based classification, thereby separating crops and weeds from the background. Feature extraction followed, and a random forest classifier was used for classification. In a separate study [8], excess green (ExG) and Otsu thresholding were employed to separate soil from weeds, followed by support vector data description (SVDD) to classify weeds and crops, specifically for maize. While these handcrafted approaches offer advantages such as low computational cost and fast processing, they generally exhibit lower accuracy in complex environments and may struggle to distinguish a wide variety of weed types.

#### 1.1.2 Deep feature-based methods

More recently, deep learning techniques utilizing convolutional neural networks (CNNs) have been widely adopted for crop and weed segmentation. Some studies have applied existing segmentation models, while others have proposed new models tailored to specific crop and weed datasets. For instance, one study [9] applied a fully convolutional network (FCN) for pixel-level classification of maize and weeds, demonstrating potential for real-time applications. Another study [10] combined RGB and NIR data with a stem detection network based on a fully convolutional densely connected network (FC-DenseNet) [11]. Research using a rice seedling dataset [12] compared models such as FCN, U-Net, and SegNet [13] and reported that SegNet exhibited the best performance. In another study [14], images from various unmanned aerial vehicles (UAVs) were used along with RGB, NIR, and NDVI inputs, where a VGG-16–based encoder was combined with a U-Net–based decoder to enhance segmentation accuracy. Similarly, a study using diverse unmanned aerial system (UAS) datasets [16] compared FCN, U-Net, SegNet, and DeepLabv3+ [17] and found that DeepLabv3+ achieved the highest performance. A transformer-based segmentation study [19] compared various transformer [18] architectures and concluded that SegFormer [20] offered optimal performance for crop and weed segmentation. A study utilizing SegNet and ENet [21] for segmentation [22] introduced residual blocks into SegNet to reduce computational cost and used a 14-channel image—created by combining RGB images with various transformations—as input, resulting in robust performance across different imaging environments. Another study [23] proposed a multi-stage segmentation approach using a cascaded encoder–decoder network (CED-Net) that conducted segmentation in four stages to improve accuracy, whereas another approach [24] introduced a multi-task semantic segmentation convolutional neural network (MTS-CNN) using two U-Nets. In the first stage, crops and weeds were treated as a single object to separate the object area from the background; in the second stage, attention mechanisms were applied for fine-grained segmentation. Furthermore, a study [25] proposed a novel strip convolutional network (SC-Net) that enhanced performance on slender targets using strip multilevel convolution, improved feature extraction via parallel multilevel convolution, and effectively fused low-level and high-level features through attention-based feature fusion. Arun et al. [26] also proposed a reduced U-Net by halving the number of filters in each convolutional layer, which maintained segmentation accuracy while reducing computational load. Another study [27] presented a modified U-Net with simplified encoder and decoder structures that achieved significantly faster performance through architectural streamlining. Although these deep feature–based methods have demonstrated high accuracy, they do not account for information beyond the camera's FOV, limiting segmentation to the visible region.

In previous studies on crop and weed segmentation, various approaches have been explored, including numerous works on multi-modal semantic segmentation using inputs such as NIR or NDVI. Multi-modal semantic segmentation aims to enhance segmentation performance by leveraging additional modalities, and a variety of methods have been proposed in the literature. For instance, MFFENet [37] fused RGB and thermal images through multi-scale feature fusion with spatial attention to achieve robust segmentation under diverse conditions. PGDENet [38] enhanced depth information from RGB and depth (RGB-D) images, and effectively integrated the RGB and enhanced depth features to achieve accurate segmentation. MTANet [39] hierarchically fused RGB and thermal information, and incorporated boundary, binary, and semantic supervision losses to achieve high accuracy even in low-light environments. EGFNet [52] improved boundary accuracy by leveraging edge information while effectively integrating RGB and thermal inputs, and utilizing high-level semantic features for accurate segmentation. MMSMCNet [53] employed modal memory fusion and morphological multiscale assistance to effectively combine complementary information from RGB and thermal images, achieving accurate semantic segmentation in poorly light scenes. MDNet [54] utilized a Mamba-based efficient fusion module and a self-distillation strategy to improve both accuracy and efficiency in RGB-thermal dense prediction. FCDENet [55] significantly improved accuracy in RGB-D scene classification by combining contrast difference modules for low-level features, high-level semantic clustering, and wavelet-based decoding. MPMTNet-KD [56] employed multi-attention perception modules, heterogeneous orientation synthesis (HOS), and multi-layer transfer knowledge distillation to simultaneously achieve performance and efficiency. Although these researches on multi-modal semantic segmentation produce very high performances with many advantages, they cannot be considered in our task because only RGB inputs without additional data can be used in our task.

### 1.2 Segmentation considering the limited FOV

Most previous studies have applied segmentation techniques that utilize only the information within the camera's FOV. To the best of our knowledge, this study is the first to address segmentation that explicitly incorporates information from beyond the FOV. The limitation of the camera's FOV may stem from factors such as the camera's position or the lens's viewing angle. In practical agricultural settings, considering regions beyond the FOV during segmentation can significantly improve the efficiency of herbicide application. In response, we propose a novel semantic segmentation model and framework—KDOSS-Net—that integrates out-of-FOV information using a lightweight architecture, thereby outperforming previous models in both scope and computational efficiency. The model and source code have been made publicly available on GitHub [28] to enable other researchers to evaluate its performance fairly. The innovations of this study are summarized as follows:

- This study presents the inaugural segmentation approach that addresses the limited FOV issue in crop and weed segmentation. To incorporate out-of-FOV information during semantic segmentation, we propose KDOSS-Net for crops and weeds, which comprises a teacher model (OPOSS-Net: object prediction–guided image outpainting and semantic segmentation network) and a student model (SSWO-Net: semantic segmentation without outpainting network). The student model learns from the teacher model via knowledge distillation (KD) to enhance segmentation accuracy. Previous studies have primarily designed outpainting methods with the assumption that the size of occluded object is large while the shape and pattern of occluded object are not complex in general scene image. However agricultural environments are characterized by a mixture of crops and weeds which exhibit small size and the complex shape and pattern. Our KDOSS-Net is designed to effectively restore such small sized and complex occlusion in agricultural scenes while maintaining computational efficiency.
- The teacher model, OPOSS-Net, is designed to perform two tasks—image outpainting and semantic segmentation—while reducing computational complexity. It comprises three sub-networks: (1) an object prediction network that predicts object regions from the limited FOV image, (2) an image outpainting network that restores the image beyond the FOV using both the object prediction and the input image, and (3) a semantic segmentation network that performs segmentation on the restored image. This design allows the external regions to be outpainted more accurately based on the predictions of shapes and distributions of crops and weeds, providing a plant-specialized structure robust to complex occlusion patterns in agricultural environments. In the image outpainting network, enhanced U-Net is proposed based on gated and dilated convolution. Gated convolution can be effective for image restoration in case of using additional guidance data. Because the object prediction result by our 1st sub-network can act as the guidance data, the gated convolution can enhance the accuracy of our image outpainting network. The size of occluded crops and weeds is small, and dilated convolution is utilized to increase the receptive field for image outpainting network. Segmentation loss by the pretrained network is also considered to the total losses of image outpainting network, forcing the outpainting to be proceeded considering both image quality and semantic segmentation accuracy of crops and weeds. Furthermore, by adopting sequential learning for three sub-networks, OPOSS-Net reduces training complexity and enhances training stability compared to the learning in an end-to-end manner, which are confirmed by our ablation study.
- The student model, SSWO-Net, performs semantic segmentation directly without undergoing the computationally intensive image outpainting process. To reduce computational complexity further, we introduce inverted residual blocks into the encoder of our lightweight enhanced U-Net architecture, which can extract more channel-wise information of features by convolution of low complexity. Because the shapes, colors, and patterns of crops are much similar to those of weeds, more channel-wise information of features should be extracted by convolution of low complexity for the high accuracy of segmentation of crops and weeds. The proposed student model is also designed for low-resource environments and has been validated to operate effectively on embedded systems and mobile devices, such as those found in agricultural robotics.
- Although lacking a dedicated image outpainting module, the student model is trained to perform both image outpainting and semantic segmentation tasks by distilling knowledge from the teacher model. To effectively transfer this dual-task knowledge, the channels of feature maps of teacher model are expanded through nonlinear transformations by multilayered perceptron (MLP) based on 1 ​× ​1 convolution, rectified linear unit (ReLU), and 1 ​× ​1 convolution, and they are normalized in channel-wise way for distillation because class information of crops and weeds are more included in channel of features. The student model assimilates this enriched information and compresses the features to enable efficient computation. Additionally, the weed information extracted using our method is automatically linked as input to the open-source large language and vision assistant (LLaVA), which facilitates the development of a system that recommends optimal herbicide strategies tailored to the detected weed class.

A comparative summary of the proposed method and existing approaches, along with their respective strengths and limitations, is provided in Table S1 (in the Supplementary Materials). The remainder of this paper is organized as follows: Section 2 describes the proposed method, Section 3 presents the experimental results, Section 4 discusses key findings, and Section 5 concludes with a summary and directions for future research.

## 2 Materials and methods

### 2.1 Overall procedure of the proposed method

**Fig. 1:**
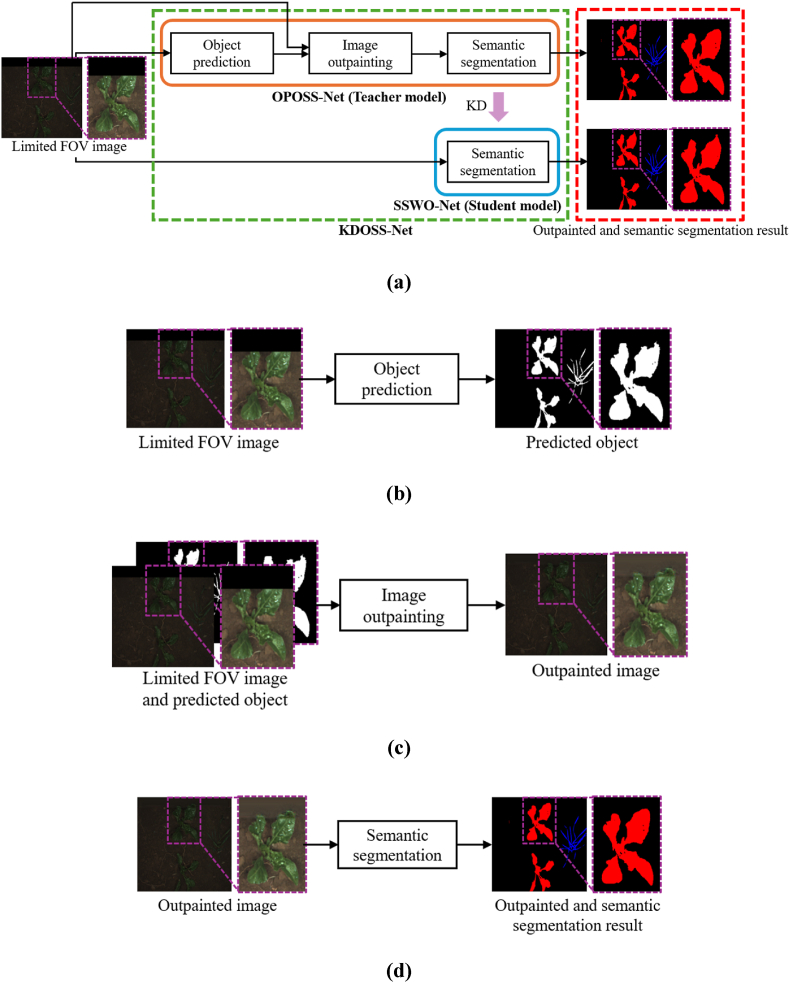
Overall procedure of proposed method (zoomed-in pictures of limited FOV and outpainted images have been brightened for visibility). (a) KDOSS-Net. (b) Object prediction network of OPOSS-Net. (c) Image outpainting network of OPOSS-Net. (d) Semantic segmentation network of OPOSS-Net. (b)–(d) represent that we perform the sequential learning of object prediction, image outpainting, and semantic segmentation networks for OPOSS-Net, respectively.

This subsection describes how the proposed KDOSS-Net processes crop and weed images with a limited field of view (FOV) to produce outpainted images and semantic segmentation results. Fig. 1 shows the overall procedure of the proposed method. When a limited FOV image is provided as input, the OPOSS-Net performs object prediction and image outpainting, followed by semantic segmentation, to generate the outpainted and segmented results. Subsequently, SSWO-Net—trained via knowledge distillation (KD) from OPOSS-Net—produces similar outpainted and semantic segmentation results without executing any explicit outpainting process.

### 2.2 KDOSS-net

This subsection describes the proposed KDOSS-Net. Section 2.2.1 explains OPOSS-Net, the teacher network in KDOSS-Net; Section 2.2.2 describes SSWO-Net, the student network; Section 2.2.3 discusses the proposed knowledge distillation strategy; and Section 2.2.4 outlines the loss function used.

#### 2.2.1 OPOSS-net (teacher network)

**Fig. 2:**
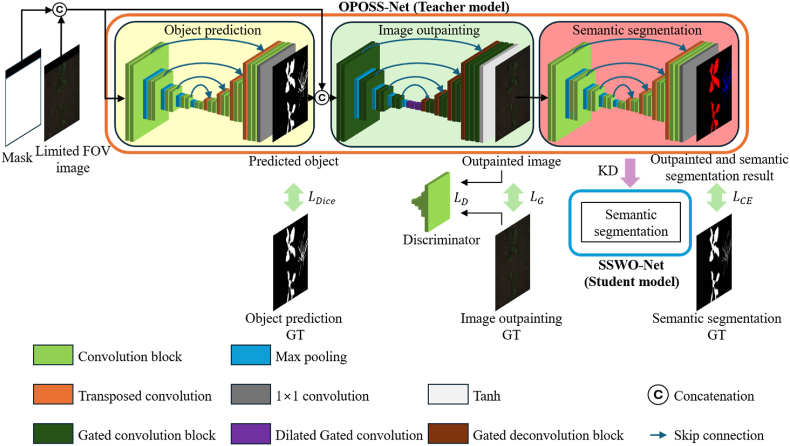
Overall structure of the proposed OPOSS-Net.

The teacher network, OPOSS-Net, consists of three components: the object prediction stage, which predicts object regions; the image outpainting stage, which generates the out-of-FOV areas using the object prediction as a guide; and the semantic segmentation stage, which performs segmentation on the outpainted image. Fig. 2 illustrates the overall structure of OPOSS-Net.

##### 2.2.1.1 Object prediction network

As shown in Fig. 2, the first sub-network of OPOSS-Net—the object prediction network—receives a limited FOV image concatenated with a mask that indicates the region to be predicted. This network then performs object prediction. Drawing inspiration from the object segmentation strategy used in MTS-CNN [24], the network is designed to infer object regions even outside the visible FOV, thereby effectively separating the foreground from the background. Unlike MTS-CNN, which focuses solely on foreground–background separation, the object prediction network in this study is trained not only to distinguish objects from the background but also to infer object information in occluded or unobservable areas beyond the FOV. The architecture of the object prediction network is based on a standard U-Net. It utilizes the limited FOV input along with segmentation targets, which are divided into foreground and background regions, to perform binary segmentation. Each convolution block uses batch normalization and the ReLU activation function. The structure of the convolution block is shown in Fig. S2(a) (Supplementary Materials). Dice loss is employed as the final loss function because it effectively captures spatial information. Eq. (1) defines the dice loss, where $P\in R^{H\times W\times Cls}$ and $L\in R^{H\times W\times Cls}$ represent the object prediction output and the ground truth label map, respectively. $P_{c}\in R^{H\times W}$ and $L_{c}\in R^{H\times W}$ denote the values corresponding to class c, and Cls indicates the number of classes—2 in this case (object and background). Note that the object class includes both crops and weeds.

(1) $$L_{Dice}=1-\frac{1}{\;Cls}\sum_{c=1}^{Cls}\frac{2\sum_{i=1}^{H\times W}P_{c}\left(i\right)\times L_{c}\left(i\right)}{\sum_{i=1}^{H\times W}\left(P_{c}\left(i\right)+L_{c}\left(i\right)\right)}$$

##### 2.2.1.2 Image outpainting network

The second sub-network of OPOSS-Net, the image outpainting network, receives as input a concatenation of the limited FOV image, a mask indicating the region to be predicted, and the output from the object prediction network. Guided by this object prediction, the network performs image outpainting. Image outpainting is a technique that generates extended visual content beyond the visible area of an image using information within the FOV. Although similar to image inpainting—which restores missing or damaged regions within an image [29]—outpainting is generally more challenging because it involves generating content in areas with significantly less contextual information. Traditional approaches to image outpainting include simple background expansion or repetition of internal patterns using classical image processing. More recent methods utilize deep generative models such as generative adversarial networks (GANs) or Transformers. Among GAN-based methods, notable examples include the semantic regeneration network (SRN) [30] and image outpainting and harmonization using GAN (IOH) [31] and generative adversarial networks for image extension (Boundless) [35]. Transformer-based approaches include U-Transformer [32]. SRN comprises a feature expansion network (FEN) for extracting features from small input images and a context prediction network (CPN) for decoding these features into complete images, taking into account padding and output dimensions. SRN enhances style consistency via context normalization and implicit diversified Markov random field (IDMRF) loss [29]. IOH, inspired by image inpainting architectures, utilizes a context encoder and is trained using a combination of reconstruction and adversarial losses. Boundless introduced semantic conditioning into the discriminator. U-Transformer integrates U-Net with the Swin Transformer [33] and introduces a temporal spatial predictor (TSP) module between the encoder and decoder to more effectively model temporal relationships between image patches, thereby facilitating more natural outpainting. However, existing image outpainting methods often struggle with structural distortion when handling complex objects and fail to preserve semantic consistency. In crop and weed datasets, the background typically dominates the image, and conventional models tend to overfit to background features, resulting in the loss of important foreground information such as crops and weeds. To address this issue, we propose an object prediction–guided image outpainting network.

The proposed image outpainting network follows a GAN architecture consisting of a generator and a discriminator. The generator is based on a U-Net structure that replaces all standard convolutions with gated convolutions [34], introduces dilated convolutions in the bottleneck (neck), and substitutes transposed convolutions for upsampling with gated deconvolutions. Gated convolution, initially developed for image inpainting, overcomes the limitations of partial convolution —which uses hard binary masks—by automatically learning soft masks and allowing for more flexible updates and better compatibility with user-guided inputs. Leveraging gated convolution, the generator can more effectively synthesize object-aware content during the outpainting process. The discriminator is based on PatchGAN and incorporates spectral normalization (SN) to enhance training stability. Detailed architectures of the gated convolution block, the gated deconvolution block, and the image outpainting network's discriminator are provided in Fig. S2(b)–(d) (Supplementary Materials).

The generator's loss function is a weighted combination of pixel reconstruction loss, adversarial loss, IDMRF loss, and cross-entropy loss. The pixel reconstruction loss, calculated using the L1 norm, compares the generated image and the ground truth image at the pixel level to ensure accurate recovery. The adversarial loss, standard in GANs, encourages the generation of high-quality images through adversarial training. The IDMRF loss evaluates feature-level consistency between the generated image and the ground truth via a pretrained CNN, enhancing visual style coherence. The cross-entropy loss uses a pretrained U-Net to produce semantic predictions from the generated image, which are then compared against the ground truth segmentation labels. These losses are defined in Eqs. (2), (3), (4), (5):

(2) $$L_{pixel}={\left|I_{gen}-I_{gt}\right|}_{1}$$

(3) $$L_{adv}={-E}_{\left(\;I_{gen}\right)}\left[D\left(I_{gen},M\right)\right]$$

(4) $$L_{IDMRF}=E_{\left(I_{gt},I_{gen}\right)}\left[IDMRF\left(V\left(I_{gen}\right),V\left(I_{gt}\right)\right)\right]$$

(5) $$L_{CrossEntropy}=-\frac{1}{H\times W}\sum_{i=1}^{H\times W}\sum_{c=1}^{Cls}L_{c}\left(i\right)\log\;P_{c}\left(i\right)$$

(6) $$L_{G}=\lambda_{pixel}L_{pixel}+\lambda_{adv}L_{adv}+\lambda_{IDMRF}L_{IDMRF}+\lambda_{CE}L_{CE}$$

In Eqs. (2), (3), (4), $I_{gen}\in R^{H\times W\times 3}$ and $I_{gt}\in R^{H\times W\times 3}$ represent the generated and ground truth images, respectively. In Eq. (3), *M* is the mask for the occluded region and *D* is the discriminator model. In Eq. (4), *V* denotes the VGG-19 [15] network pretrained on ImageNet. In Eq. (5), $P\in R^{H\times W\times Cls}$ and $L\in R^{H\times W\times Cls}$ represent the semantic segmentation results generated by the pretrained U-Net and the ground truth label map, respectively. $P_{c}\in R^{H\times W}$ and $L_{c}\in R^{H\times W}$ correspond to class c, and Cls is 3 (crop, weed, background). The pixel reconstruction loss in Eq. (2) is computed using L1 loss between the generated and ground truth images. For adversarial loss in Eq. (3), Wasserstein GAN (WGAN) [36] loss is adopted, training the generator to produce images that receive higher scores from the discriminator. The IDMRF loss in Eq. (4), adapted from prior image outpainting work, maintains perceptual similarity and texture clarity by comparing cosine similarity of multilevel features extracted from VGG-19. The cross-entropy loss in Eq. (5) compares semantic predictions derived from the generated image to ground truth labels, allowing the image outpainting process to assist in downstream segmentation tasks. The final generator loss function is given in Eq. (6), where the weights $\lambda_{pixel}$, $\lambda_{adv}$, $\lambda_{IDMRF}$, and $\lambda_{CE}$ are empirically set to 1, 0.1, 0.01, and 0.01, respectively, based on the best semantic segmentation accuracy observed during training. Although we performed the intensively comparative experiments with more combination of $\lambda_{pixel}$, $\lambda_{adv}$, $\lambda_{IDMRF}$, and $\lambda_{CE}$, we included only the results from representative 4 cases of combinations in Table S14 (Supplementary Materials) for the simplicity of table. Considering the highest segmentation accuracy in terms of *mIOU* and *F*1 *score* as shown in this table, which are the final goal of our research, we determined the optimal combination of $\lambda_{pixel}$, $\lambda_{adv}$, $\lambda_{IDMRF}$, and $\lambda_{CE}$ in our experiment.

For the discriminator, the WGAN-GP [36] loss is adopted, which includes a gradient penalty (GP) to enforce the 1-Lipschitz constraint. This penalty encourages smoother gradients and more stable training by penalizing deviations of the gradient norms from unity. The GP term and the final discriminator loss are defined in Eqs. (7), (8). In Eq. (7), $\hat{I}$ represents a sample interpolated between $I_{gt}$ and $I_{gen}$, which is used to compute the gradient penalty. The discriminator is trained to assign higher scores to real images and lower scores to generated (fake) images while ensuring Lipschitz continuity through GP. In Eq. (8), $\lambda_{GP}$—the weight for $L_{GP}$—is empirically set to 20, based on the best observed semantic segmentation performance.

(7) $$L_{GP}={-E}_{\left(\hat{I}\right)}\left[{\left({\left\|\nabla_{\hat{I}}D\left(\hat{I},M\right)\right\|}_{2}-1\right)}^{2}\right]$$

(8) $$L_{D}={E_{\left(I_{gt}\right)}\left[D\left(I_{gt},M\right)\right]-E}_{\left(I_{gen}\right)}\left[D\left(I_{gen},M\right)\right]+\lambda_{GP}L_{GP}$$

##### 2.2.1.3 Semantic segmentation network

The final component of OPOSS-Net is the semantic segmentation network, which takes the outpainted image—with the extended area beyond the original FOV—as input and generates the final semantic segmentation result. This network utilizes a U-Net architecture—the highest performing baseline model in the ablation study of Subsection 3.2.1.4. It detects crop and weed regions in the restored image, and the loss function used is the cross-entropy loss as defined in Eq. (5). Here, $P\in R^{H\times W\times Cls}$ and $L\in R^{H\times W\times Cls}$ represent the semantic segmentation result and the ground truth segmentation label map, respectively. $P_{c}\in R^{H\times W}$ and $L_{c}\in R^{H\times W}$ refer to the values corresponding to class c, and the number of classes Cls for this network is 3: crop, weed, and background.

#### 2.2.2 SSWO-net (student network)

**Fig. 3:**
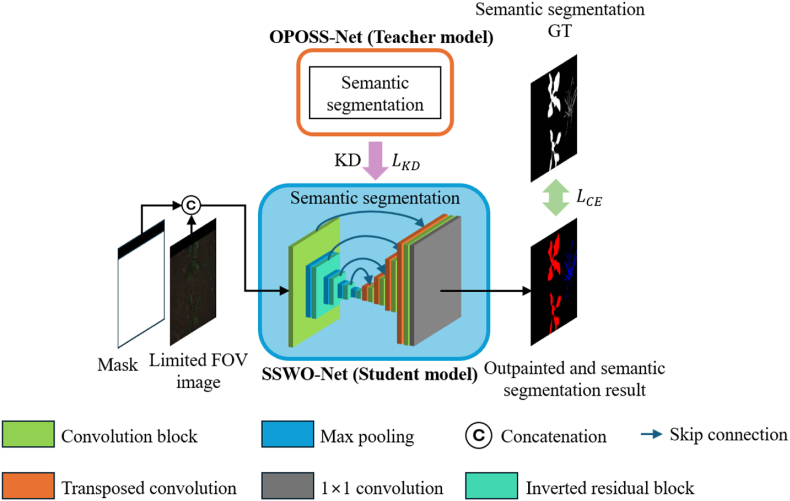
Overall structure of proposed SSWO-Net.

This subsection describes SSWO-Net, the student network used in this study. SSWO-Net receives as input the concatenation of the limited FOV image and the mask for the out-of-FOV area. It comprises a single network that directly produces the outpainted image and the semantic segmentation result. SSWO-Net is trained using knowledge distillation (KD) from the semantic segmentation network of OPOSS-Net to enhance performance. Fig. 3 illustrates the overall architecture of SSWO-Net.

Unlike the teacher network OPOSS-Net, which includes a restoration process, SSWO-Net performs only semantic segmentation—without explicit image outpainting—while still generating results comparable to those produced by outpainted segmentation. The structure of SSWO-Net is derived from U-Net, with a key modification: all encoder blocks, except for the initial convolution block, are replaced with inverted residual blocks [40] to significantly reduce the number of parameters. The inverted residual block, originally proposed in MobileNetV2 [40], combines depth-wise separable convolutions with bottleneck structures to facilitate efficient computation and model compression. It first expands the dimensionality of the input feature map using a (1 ∖times 1) convolution, then applies depth-wise convolution—where separate filters are applied independently to each channel—to reduce computational cost, and finally uses a second (1 ∖times 1) convolution to fuse the output channels. A residual connection is added by merging the input feature map with the output, allowing for improved gradient flow and performance. The detailed architecture of the inverted residual block is shown in Fig. S2(e) (Supplementary Materials). In the decoder, the number of convolution blocks per stage is reduced from two (as in the standard U-Net) to one, further decreasing the number of parameters and computational cost.

#### 2.2.3 Knowledge distillation (KD)

**Fig. 4:**
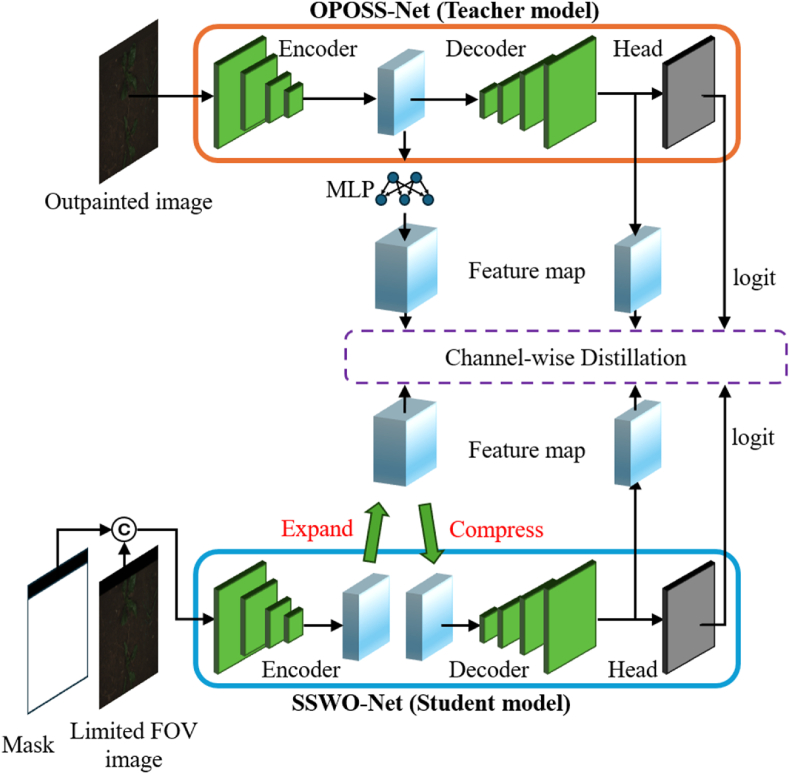
Proposed KD methods.

This subsection describes the knowledge distillation (KD) method proposed in this study. Prior research on KD can be broadly categorized into logit-based and feature-based approaches. Logit-based methods, such as the study by Ba and Caruana [41], demonstrated that even shallow networks can improve in performance through distillation. Subsequently, Hinton et al. [5] proposed minimizing the Kullback-Leibler (KL) divergence between the teacher's and student's logits to implement soft-target–based KD. In feature-based KD, FitNet [42] introduced the use of intermediate feature maps, an approach that was further developed by methods such as attention transfer (AT) [43], feature-wise similarity preserving (FSP) [44], and similarity preserving (SP) [45]. In the context of semantic segmentation, channel-wise distillation (CWD) [46] was proposed, where the activation maps of individual channels are normalized into soft probability maps and KD is performed by minimizing the KL divergence between these maps. A recent study [47] proposed an MLP-based channel-wise transformation to align the feature maps of teacher and student networks through learnable nonlinear mappings, while another study [48] emphasized the importance of projectors when significant architectural differences exist between the teacher and student networks. Additionally, Attention-Guided Feature Distillation [49] employed the convolutional block attention module (CBAM) to generate feature maps that integrate both channel and spatial information prior to distillation. Building on these approaches, we propose a KD strategy tailored to SSWO-Net. The proposed KD framework is illustrated in Fig. 4.

As shown in Fig. 4, KD is applied at three key locations: the final feature map of the encoder (referred to as “mid”), the final feature map of the decoder (referred to as “last”), and the semantic segmentation logits (referred to as “logit”). Applying KD at the encoder's final feature map allows the student network to absorb rich channel-wise representations; applying KD at the decoder's final feature map transfers fine-grained spatial information; and applying KD at the logits level promotes consistency in the final classification results, thus improving overall segmentation accuracy. For channel-wise distillation, the probability map for each channel is normalized, and the KL divergence is minimized between corresponding teacher and student maps. This approach enables the student model to more effectively learn from the teacher's important channels by incorporating per-channel importance weighting into the loss function [46]. To further enhance knowledge transfer, we adopt a bottleneck-style structure—similar to that in MobileNetV2—that expands and then compresses feature maps, thereby enriching the information transferred between the teacher and student. This structure is applied during distillation at the “neck” level of both networks. The MLP module shown in Fig. 4 follows the design in Ref. [47], consisting of a 1 ​× ​1 convolution, a ReLU activation, and another 1 ​× ​1 convolution, serving as a learnable nonlinear channel-wise transformation. Unlike [47], in our design the teacher's feature map channels are expanded to ensure more effective transfer of information to the student.

With this design, our KD strategy is optimized for SSWO-Net. The details of channel-wise distillation are defined in Eqs. (9), (10), (11), and the final KD loss is defined in Eq. (12). In Eq. (9), the channel-wise normalization process is described using a temperature parameter Τ set to 4. Eq. (10) defines the KL divergence. In Eq. (11), $F^{T}\in R^{H\times W\times C}$ and $F^{S}\in R^{H\times W\times C}$ denote the feature maps from the teacher and student models, respectively. Finally, Eq. (12) defines $L_{KD}$, the total KD loss, as the sum of the losses from the three distillation locations. Here, $F_{mid}^{T},F_{mid}^{S},F_{last}^{T},F_{last}^{S}$ denote the teacher and student feature maps at the encoder and decoder outputs, and $F_{logit}^{T},F_{logit}^{S}$ represent the final semantic segmentation logits. The teacher's mid-level feature map $F_{mid}^{T}$ is expanded using the nonlinear channel-wise transformation from the MLP to align with the student's expanded feature map $F_{mid}^{S}$ during distillation.

(9) φ(yc)=exp⁡(yicΤ)∑i=1H×Wexp⁡(yicΤ)

(10) $$KL\left(P_{c}^{T},P_{c}^{S}\right)=\sum_{i=1}^{H\times W}P_{c}^{T}\left(i\right)\log\frac{P_{c}^{T}\left(i\right)}{P_{c}^{S}\left(i\right)}$$

(11) $$CWD\left(F^{T},F^{S}\right)=\frac{{Τ}^{2}}{C}\sum_{c=1}^{C}KL\left(\varphi \left(F_{c}^{T}\right),\varphi \left(F_{c}^{S}\right)\right)$$

(12) $$L_{KD}=CWD\left(MLP\left(F_{mid}^{T}\right),F_{mid}^{S}\right)+CWD\left(F_{last}^{T},F_{last}^{S}\right)+CWD\left(F_{logit}^{T},F_{logit}^{S}\right)$$

#### 2.2.4 Loss function

This subsection describes the final loss function used to train the student model, SSWO-Net, within KDOSS-Net. The final loss function, defined in Eq. (13), is a combination of the cross-entropy loss with the ground truth segmentation labels and the KD loss distilled from OPOSS-Net. The weighting factor for the KD loss, $\lambda_{KD}$, was determined empirically based on the best semantic segmentation accuracy for each dataset: 0.3 for the rice seedling and weed dataset, 0.2 for the BoniRob dataset, and 0.1 for the crop/weed field image dataset (CWFID).

(13) $$L_{total}=\left(1-\lambda_{KD}\right)L_{CE}+\lambda_{KD}L_{KD}$$

### 2.3 Experimental dataset and setup

The experiments were conducted using three public datasets: the CWFID dataset [50], the BoniRob dataset [51], and the rice seedling and weed dataset [12]. Each dataset comprises crop and weed images along with ground truth label pairs. The CWFID dataset includes ground truth labels for carrots and weeds, and a JAI AD-130GE camera was utilized to acquire the images of 1296 ​× ​966 pixels. The BoniRob dataset have images acquired by a camera on farming robot, which contains the classes for sugar beet plants, grass weeds, and dicot weeds. We used the images of 1296 ​× ​966 pixels with the classes of sugar beet plants and grass weeds. The rice seedling and weed dataset was acquired by an IXUS 1000 HS camera (with an EF-S 36–360 ​mm lens of f/3.4–5.6), and the spatial resolution of images is 912 ​× ​1024 pixels, with classes of rice and Sagittaria trifolia weed. All experiments were performed using 2-fold cross-validation, ensuring no overlap between training and testing datasets in each fold, with approximately 11 ​% of the training data used for validation. For training KDOSS-Net, all datasets were resized to 512 ​× ​512 pixels using bilinear interpolation, and spatial data augmentation (flip, rotation) was applied. Examples of the public crop and weed datasets are provided in Fig. S3 (Supplementary Materials), and Table S2 summarizes the number of images per dataset and fold. All experiments were performed on a desktop running Windows 11, equipped with an AMD Ryzen™ 7 7800X3D 8-Core Processor, 64 ​GB RAM, and an NVIDIA GeForce RTX 4080 SUPER. The proposed method was implemented using PyTorch 2.5.1.

### 2.4 Limited FOV datasets for experiments

To train and evaluate the outpainting and semantic segmentation models for crop and weed images, it was necessary to prepare limited field-of-view (FOV) images. Generating ground truth segmentation labels for crops and weeds typically requires expert annotation, making it challenging to build a self-collected limited FOV dataset. Moreover, no existing public datasets simultaneously provide limited FOV images and crop/weed segmentation labels. Consequently, in this study, limited FOV datasets were constructed from the CWFID, BoniRob, and rice seedling and weed datasets. Rather than simply occluding a fixed portion of each image—which may only obscure the background—the method selectively masks the upper 10 ​% of the crop and weed regions based on the regions of interest (ROI) in the segmentation ground truth. This approach ensures that the occluded regions contain meaningful object information, rendering the restoration task more relevant. The constructed limited FOV datasets have been uploaded to the project's GitHub repository [28].

### 2.5 Evaluation metrics

To evaluate the performance of both the outpainting and semantic segmentation methods, the following metrics were employed: *Accuracy*, intersection over union (*IOU*) for crops and weeds, mean intersection over union (*mIOU*), *Recall*, *Precision*, and *F1 score*. These metrics are defined in Eqs. (14), (15), (16), (17), (18), (19). *Accuracy* is calculated as the proportion of correctly predicted pixels over the total number of predictions. For *mIOU*, as in prior studies such as CED-Net [23] and MTS-CNN [24], the background class is excluded and only the IoU values for crops and weeds are averaged (micro average); accordingly, in Eq. (16), the number of classes Cls is set to 2 (crop and weed only). *Recall* measures the ratio of correctly predicted positive pixels to all actual positive pixels, indicating how well relevant objects are detected. *Precision* measures the ratio of correctly predicted positive pixels to all pixels predicted as positive, reflecting the model's ability to avoid false positives. The *F1 score*, calculated as the harmonic mean of precision and recall, provides a balanced assessment of overall performance.

(14) $$Accuracy=\frac{TP+FP}{TP+TN+FN+FP}$$

(15) $$IOU=\frac{TP}{TP+FN+FP}$$

(16) $$mIOU=\frac{\sum_{i=1}^{Cls}TP_{i}}{\sum_{i=1}^{Cls}\;TP_{i}+FN_{i}+FP_{i}}$$

(17) $$Recall=\frac{TP}{TP+FN}$$

(18) $$Precision=\frac{TP}{TP+FP}$$

(19) $$F1\;score=\frac{2\times precision\times recall}{precision+recall}$$

## 3 Experimental results

### 3.1 Model training

Both OPOSS-Net and SSWO-Net proposed in this paper use the adaptive moment estimation (Adam) optimizer. The detailed hyperparameter settings for each model used in KDOSS-Net are provided in Table S3 (Supplementary Materials). Fig. S4 (Supplementary Materials) shows the changes in training and validation losses, as defined Eq. (13), across epochs during the training of the final student model in KDOSS-Net. The training loss converged to a sufficiently low value, with a steep initial drop followed by a more gradual decline as training progressed. This indicates that the proposed model was effectively trained on the training data. The validation loss also converged to a low value as the number of epochs increased and did not exhibit significant increases in the later stages of training, suggesting that the model did not overfit the training data. Figs. S5 and S6 (Supplementary Materials) show the evolution of the cross-entropy loss and KD losses (mid, last, and logit), as defined in Eqs. (12), (13), during training and validation. In the early training stages, the cross-entropy loss decreased rapidly, while the KD losses declined more gradually. In the later stages, all losses, including the KD losses, converged to small values, indicating that the model initially focused on learning the segmentation task. Furthermore, the logit values distilled after the segmentation head converged earlier than the final feature maps distilled from the decoder's last layer. This is likely because the segmentation head refines features to generate class-discriminative logits, which are more directly tied to the segmentation task and therefore converge faster than the decoder's final feature maps. When comparing training and validation losses, the validation loss showed a relatively smaller decrease, but all losses still sufficiently converged. This indicates that the knowledge from the teacher model was gradually transferred to the student model, even on validation data.

### 3.2 Testing of proposed method

#### 3.2.1 Ablation studies

In this study, ablation experiments were conducted from three perspectives: First, to examine whether image outpainting in the teacher network, OPOSS-Net, meaningfully affects semantic segmentation performance. Second, to evaluate the contribution of each stage in the three-stage architecture of OPOSS-Net. Third, to analyze the impact of knowledge distillation from OPOSS-Net to SSWO-Net based on the location of the feature maps used for distillation.

##### 3.2.1.1 Effect of image outpainting on semantic segmentation performance under limited FOV

First, we compared the semantic segmentation performance of U-Net when the teacher model was used for limited FOV image restoration. U-Net was selected for this analysis because, as shown in Subsection 3.2.1.4, it exhibited the best performance among the tested segmentation models. The results of each scheme are presented in Table 1. In Table 1, comparing the baseline case in which both training and testing were conducted on the original image using U-Net (Scheme 1), a decrease of 8.8 ​% in *mIOU* was observed when the model trained on original images was tested on a limited FOV dataset (Scheme 2). However, when the image was restored through outpainting and then tested (Scheme 4), a 1.6 ​% improvement in *mIOU* was observed. Furthermore, comparing the case where both training and testing were conducted on a limited FOV dataset (Scheme 3) with the case where training and testing were performed on outpainted images (Scheme 5), a 2.02 ​% improvement was achieved. These results demonstrate that the image outpainting method proposed in this paper improves semantic segmentation performance in cases where a limited FOV reduces accuracy. In other words, the ablation studies presented in this paper highlight how incorporating information from outside the FOV in OPOSS-Net contributes to performance enhancement in semantic segmentation.

**Table 1:** Comparisons of semantic segmentation accuracies according to original data, limited FOV data, and image outpainting by proposed OPOSS-Net.

Methods	*Accuracy*	mIOU	Crop IOU	Weed IOU	Recall	Precision	F1score
Scheme 1 (baseline)	0.9901	0.8219	0.8744	0.6293	0.8946	0.9055	0.8994
Scheme 2	0.9849	0.7339	0.7675	0.5645	0.7906	0.9072	0.8411
Scheme 3	0.9862	0.7506	0.7897	0.5611	0.8102	0.9059	0.8523
Scheme 4	0.9859	0.7499	0.7861	0.5653	0.8178	0.8956	0.8519
Scheme 5 (proposed)	**0.9873**	**0.7708**	**0.8063**	**0.5934**	**0.8320**	**0.9086**	**0.8659**

**Scheme 1**: Training and testing of U-Net with original data without limited FOV.

**Scheme 2**: Training of U-Net with original data without limited FOV and testing of U-Net with limited FOV data.

**Scheme 3**: Training and testing of U-Net with limited FOV data.

**Scheme 4**: Training of U-Net with original data without limited FOV and testing of U-Net with data restored by OPOSS-Net.

**Scheme 5**: Training and testing of U-Net with data restored by OPOSS-Net.

##### 3.2.1.2 Effect of object prediction and image outpainting network in OPOSS-Net

In this subsection, an ablation study was conducted to evaluate the effects of the object prediction network and the image outpainting network, which support the final semantic segmentation network in OPOSS-Net. For this experiment, the final semantic segmentation network was kept fixed, and different cases were considered based on the presence or absence of the object prediction and image outpainting networks. A summary of these cases is provided in Table S4 (Supplementary Materials), and their respective performances are shown in Table 2. As shown in Table 2, the proposed method, which employs all three subnetworks, achieved the highest *mIOU* performance. The lowest *mIOU* (0.7506) was observed in the case where only the semantic segmentation network was used (Case 1). Incorporating object prediction before semantic segmentation (Case 2) led to a 0.57 ​% improvement in *mIOU* over Case 1. Similarly, performing semantic segmentation after image outpainting (Case 3) resulted in a 0.76 ​% improvement compared to Case 1. These results suggest that the image outpainting network contributes more significantly to accuracy improvements than the object prediction network. When all subnetworks were used (Case 4), the *mIOU* improved by 2.02 ​% compared to Case 1, showing the greatest overall performance enhancement. In other words, in OPOSS-Net, the processes of object prediction and image outpainting facilitate the recovery of information outside the FOV, thereby enhancing semantic segmentation performance relative to using only the semantic segmentation network.

**Table 2:** Comparative accuracies of semantic segmentation according to three subnetworks of OPOSS-Net.

Methods	*Accuracy*	mIOU	Crop IOU	Weed IOU	Recall	Precision	F1score
Case 1	0.9862	0.7506	0.7897	0.5611	0.8102	0.9059	0.8523
Case 2	0.9867	0.7563	0.7904	0.5682	0.8146	0.9086	0.8559
Case 3	0.9867	0.7582	0.7950	0.5760	0.8146	**0.9112**	0.8572
Case 4 (proposed)	**0.9873**	**0.7708**	**0.8063**	**0.5934**	**0.8320**	0.9086	**0.8659**

##### 3.2.1.3 Effect of proposed outpainting method on semantic segmentation performance

In this subsection, we compare the image quality and semantic segmentation results after image outpainting using OPOSS-Net with those obtained using other state-of-the-art (SOTA) image outpainting networks. Image quality was evaluated using the peak signal-to-noise ratio (*PSNR*) and the structural similarity index measure (*SSIM*), as defined in Eqs. (20), (21). A higher *PSNR* indicates a closer similarity to the original image, while *SSIM*, based on human visual perception, measures structural similarity through luminance, contrast, and structure components. A value closer to 1 indicates a higher structural similarity between the two images. In Eq. (20), $I_{gen}$ and $I_{gt}$ represent the image generated by the generator and the ground truth image, respectively. “Max” refers to the maximum pixel value that can be obtained in the image, and the mean square error (MSE) is the average squared error between the pixel values of the original image and the outpainted image. In Eq. (21), $\mu_{x}$ and $\mu_{y}$ are the means of images *x* and *y*, $\sigma_{x}^{2}$ and $\sigma_{y}^{2}$ are the variances, and $\sigma_{xy}$ is the covariance. The constants m and n are used to stabilize the formula.

(20) $$PSNR=10\;\log_{10}\left(\frac{{\mathrm{Max}\left(I_{gen}\right)}^{2}}{MSE\left(I_{gt},I_{gen}\right)}\right)$$

(21) $$SSIM\left(x,y\right)=\frac{\left(2\mu_{x}\mu_{y}+m^{2}\right)\left(2\sigma_{xy}+n^{2}\right)}{\left(\mu_{x}^{2}+\mu_{y}^{2}+m^{2}\right)\left(\sigma_{x}^{2}+\sigma_{y}^{2}+n^{2}\right)}$$

**Fig. 5:**
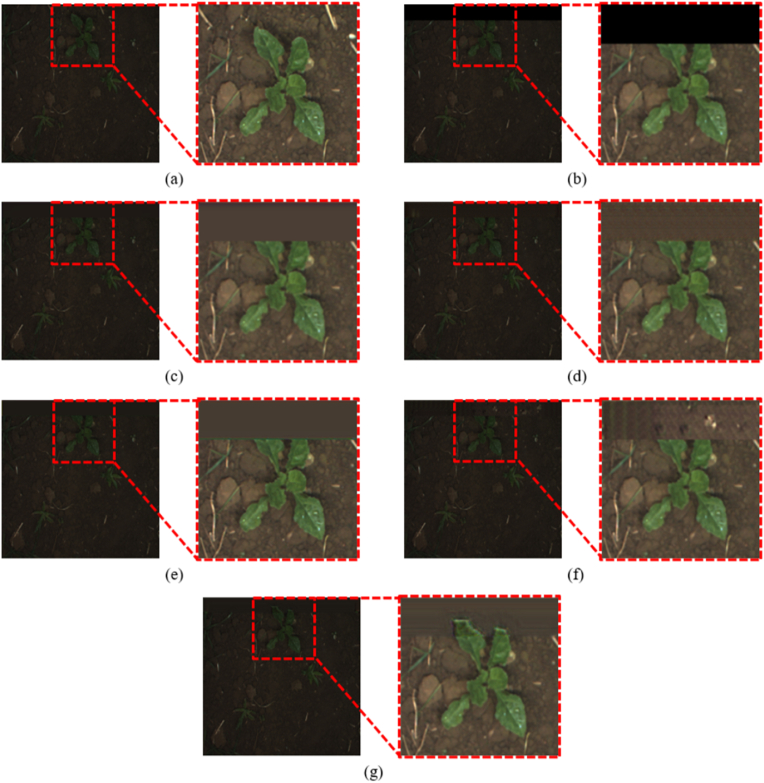
Examples of restored images using SOTA outpainting models and OPOSS-Net. (a) Original image, (b) limited FOV image, outpainted images by (c) SRN, (d) IOH, (e) Boundless, (f) U-transformer, and (g) proposed OPOSS-Net (Zoomed-in pictures of each outpainted image have been brightened for visibility).

For fair comparisons, we used U-Net as semantic segmentation for all the cases. The performance for each case is presented in Table 3, and the visual comparison of the images is shown in Fig. 5. In the case of other image outpainting networks, due to the characteristics of the crop and weed dataset, where the background occupies a large portion of the image, most of the generated areas were often background. This led to a slight improvement compared to using only the semantic segmentation network without the image outpainting process (as seen in Case 1 of Table 2, where training and testing were performed on a limited FOV dataset). However, it did not significantly contribute to restoring information outside the field of view. In contrast, the proposed OPOSS-Net performed outpainting based on object prediction values, allowing it to focus more on the crop and weed areas compared to other models. As a result, OPOSS-Net achieved the best performance in semantic segmentation. In terms of image quality, SRN achieved the highest *PSNR* and *SSIM* performance, with U-transformer also surpassing the proposed method in terms of these metrics. Although the proposed method showed relatively lower performance in *PSNR* and *SSIM* compared to other methods, it was more effective in preserving the semantic features essential for the semantic segmentation task. As a result, the proposed method was able to achieve superior performance in semantic segmentation accuracy, which was the primary goal of this study.

**Table 3:** Comparison of image quality and semantic segmentation results after restoration using OPOSS-Net and various image outpainting networks.

Methods	*PSNR*	*SSIM*	*Accuracy*	mIOU	Crop IOU	Weed IOU	Recall	Precision	F1score
SRN [30]	**42.11**	**0.9829**	0.9865	0.7562	0.7924	0.5671	0.8195	0.9016	0.8559
IOH [31]	38.79	0.9667	0.9865	0.7560	0.7950	0.5647	0.8196	0.9007	0.8557
Boundless [35]	39.67	0.9780	0.9866	0.7569	0.7941	0.5708	0.8160	0.9061	0.8560
U-transformer [32]	40.97	0.9761	0.9864	0.7571	0.7954	0.5729	0.8209	0.9015	0.8567
Proposed	39.97	0.9691	**0.9873**	**0.7708**	**0.8063**	**0.5934**	**0.8320**	**0.9086**	**0.8659**

As shown in the examples of the restored images using each outpainting model in Fig. 5, unlike other methods that focus on generating the background, the proposed OPOSS-Net focuses more on the crop and weed areas. This demonstrates that the proposed method, which performs object prediction first and then uses the predicted values as a guideline for outpainting, can focus more on the object regions, even in situations with a large amount of background and complex objects, compared to existing methods including transformer-based and Boundless for outpainting methods.

##### 3.2.1.4 Effect of proposed outpainting method across different segmentation models

In this subsection, we present a comparison utilizing different semantic segmentation models in OPOSS-Net's semantic segmentation network. For the experiment, images were first restored using the proposed object prediction and image outpainting method, and then semantic segmentation was performed using different models. To demonstrate that the object prediction and image outpainting processes contribute to performance improvement across all semantic segmentation models, the results of performing semantic segmentation without object prediction and image outpainting are also provided in Table S5 (Supplementary Materials). Among the four models, U-Net showed the best performance, so U-Net was chosen as the final semantic segmentation network for OPOSS-Net. Not only for U-Net, but also for other models, performance improved when object prediction and image outpainting were performed before segmentation, compared to when segmentation was trained without these processes. This shows that the proposed method helps to improve semantic segmentation performance by reflecting the area outside the FOV for each semantic segmentation network, not only for U-Net.

##### 3.2.1.5 Effect of sequential or end-to-end learning on semantic segmentation performance

In this subsection, the impact of sequential learning on semantic segmentation performance and training time is analyzed when training the teacher model, OPOSS-Net. In Table S6 (Supplementary Materials), the semantic segmentation accuracy and total training time for OPOSS-Net are measured and presented when trained using end-to-end learning and sequential learning. In the case of sequential learning, not only was the training time shorter than that of end-to-end learning, but the stability of the training in both the restoration network and the segmentation network was improved, leading to an enhancement in semantic segmentation accuracy as well. This demonstrates that, in this study, sequential learning is more suitable than end-to-end learning.

##### 3.2.1.6 Effect of KD on semantic segmentation performance

In this subsection, the impact of applying KD on semantic segmentation performance when training the student model, SSWO-Net, is analyzed. In Table S7 (Supplementary Materials), the performance of the teacher model, OPOSS-Net, the standalone performance of SSWO-Net trained without KD, and the performance of the student model when the proposed KD is applied are presented. In Table S7 (Supplementary Materials), it can be observed that applying KD to SSWO-Net resulted in a 2.16 ​% improvement in *mIOU* compared to when KD was not applied.

##### 3.2.1.7 Effect of channel expansion in KD

In this subsection, the impact of channel expansion on KD performance when applying the proposed KD method at the last part of the encoder is analyzed. Keeping all other conditions the same, the performance of distilling the mid feature map without channel expansion and with channel expansion is presented in Table S8 (Supplementary Materials). In Table S8 (Supplementary Materials), when KD was performed after increasing the teacher model's channel number with channel expansion, there was a 0.88 ​% improvement in *mIOU* compared to when KD was performed without channel expansion. This shows that increasing the teacher model's channels during KD to distill richer information helps the student model better follow the channel representations of the teacher model.

##### 3.2.1.8 Effect of MLP in KD

In this subsection, we analyze the effect of applying a nonlinear channel transformation MLP during knowledge distillation (KD) with channel expansion at the final part of the encoder. Two methods for distilling mid-level feature maps are compared: one uses only a 1 ​× ​1 convolution (Linear), while the other employs a 1 ​× ​1 convolution followed by a ReLU activation and another 1 ​× ​1 convolution (MLP). The results of this comparison are provided in Table S9 (Supplementary Materials). As shown, using the MLP structure leads to better performance than using the Linear approach. This indicates that introducing nonlinearity into the channel matching process, as also demonstrated in prior research [47], can improve the effectiveness of knowledge distillation.

##### 3.2.1.9 Effect of location of KD loss application

This subsection presents an ablation study on the effect of different KD loss application locations within the model. Three types of KD losses used in this study were individually and jointly examined. The experimental settings for each case are summarized in Table S10 (Supplementary Materials), and the performance results are reported in Table 4. As shown in Table 4, applying any form of KD loss led to performance improvements compared to the baseline without KD (Case 1). Among the single-loss cases (Cases 2–4), Case 2, which applied KD to the logits only, achieved the highest performance gain, showing a 0.94 ​% improvement in *mIOU* over Case 1. This suggests that distillation from the model's final output (logits) is more effective than feature-based distillation that relies solely on intermediate feature maps. Moreover, combining multiple KD losses led to better performance than using individual losses. In Case 5, where both mid-level features and logits were distilled, the model achieved the second-best results in terms of *mIOU* and F1 score, following Case 8, which used all three proposed losses. This is likely because the mid-level features and logits are spatially and semantically close in the network, resulting in complementary information being distilled. Furthermore, comparing Case 6 (without mid-level KD) and Case 8 (with mid-level KD) shows that including mid-level feature distillation also contributes to improved final segmentation performance.

**Table 4:** Comparison of applying KD at three different locations in the student model (SSWO-Net).

Methods	*Accuracy*	mIOU	Crop IOU	Weed IOU	Recall	Precision	F1score
Case 1	0.9806	0.7308	0.7741	0.5311	0.8014	0.8850	0.8380
Case 2	0.9854	0.7402	0.7752	0.5577	0.8123	0.8868	0.8450
Case 3	0.9844	0.7383	0.7792	0.5376	0.8055	0.8929	0.8434
Case 4	0.9854	0.7393	0.7832	0.5460	0.8106	0.8872	0.8443
Case 5	0.9860	0.7501	0.7887	0.5678	0.8126	0.9021	0.8519
Case 6	0.9860	0.7438	0.7854	0.5521	0.7976	**0.9122**	0.8473
Case 7	0.9858	0.7447	0.7817	0.5624	0.8092	0.8969	0.8479
Case 8 (proposed)	**0.9861**	**0.7524**	**0.7905**	**0.5735**	**0.8225**	0.8940	**0.8540**

#### 3.2.2 Comparisons of semantic segmentation accuracy by proposed and SOTA methods

In this subsection, the performance of the proposed KDOSS-Net is compared with state-of-the-art (SOTA) segmentation methods across three datasets: Rice seedling and weed, CWFID, and BoniRob. All models were trained under the same experimental conditions, using the same training epochs, batch size, and other hyperparameters as those used for SSWO-Net.

##### 3.2.2.1 Rice seedling and weed dataset

**Fig. 6:**
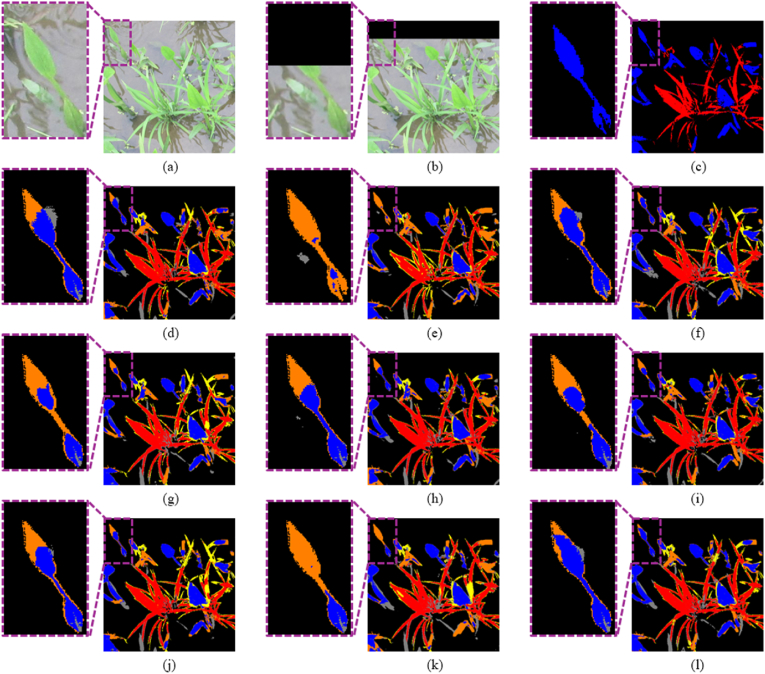
Comparison of semantic segmentation results between SOTA methods and KDOSS-Net on the Rice seedling and weed dataset. Red, blue, and black indicate crops, weeds, and background, respectively. Yellow represents errors of crops being incorrectly identified as weeds or background whereas orange means errors of weeds being incorrectly detected as crops or background. Gray represents errors of the background being incorrectly identified as crops or weeds. (a) Original image; (b) limited FOV image; (c) ground truth label. Result images by (d) U-Net, (e) Deeplabv3+, (f) SegNet, (g) FCN-8s, (h) reduced U-Net, (i) modified U-Net, (j) SC-Net, (k) Segformer-B0, and (l) KDOSS-Net (proposed methods).

Table 5 presents the quantitative comparison results, and Fig. 6 provides a qualitative comparison of semantic segmentation outputs between SOTA methods and KDOSS-Net. As seen in Table 5, the proposed method achieves the highest performance across all metrics, including *Accuracy*, *mIOU*, Crop *IOU*, Weed *IOU*, *Precision*, and *F1 score*. Compared to SegNet—the second-best performing model in terms of *mIOU*—KDOSS-Net outperforms it by 1.54 ​%. While the proposed method yields a lower *Recall* than SegNet, this is attributed to SegNet's tendency to classify a broader area as the positive class, capturing more true positives. However, this also increases false positives due to misclassification of background areas, resulting in lower *Precision*. Consequently, despite its higher *Recall*, SegNet records a lower *F1 score* than the proposed method. As shown in the zoomed-in regions of Fig. 6, other comparison models fail to detect crops and weeds in the out-of-FOV areas, leading to significant errors. In contrast, the proposed model produces fewer errors and successfully identifies crops and weeds even beyond the FOV. Furthermore, it performs comparably to other methods within the in-FOV areas. These results demonstrate that the proposed model not only performs well within the FOV but also achieves superior segmentation performance in out-of-FOV regions, effectively capturing detailed information of occluded objects that result from the limited FOV.

**Table 5:** Comparative accuracies of semantic segmentation on rice seedling and weed dataset with SOTA segmentation models and proposed KDOSS-Net.

Methods	*Accuracy*	mIOU	Crop IOU	Weed IOU	Recall	Precision	F1score
U-Net [14]	0.9307	0.6126	0.5942	0.6059	0.7750	0.7494	0.7556
Deeplabv3+ [16]	0.9249	0.5905	0.5733	0.5827	0.7267	0.7636	0.7402
SegNet [12]	0.9264	0.6161	0.5956	0.6264	**0.8129**	0.7238	0.7585
FCN-8s [9]	0.9268	0.5989	0.5803	0.5948	0.7637	0.7391	0.7452
Reduced U-Net [26]	0.9286	0.6049	0.5871	0.5934	0.7752	0.7373	0.7498
Modified U-Net [27]	0.9278	0.6078	0.5894	0.6039	0.7946	0.7243	0.7518
SC-Net [25]	0.9256	0.5952	0.5783	0.5873	0.7714	0.7268	0.7408
Segformer-B0 [19]	0.9226	0.5778	0.5682	0.5490	0.7652	0.7031	0.7268
KDOSS-Net (proposed)	**0.9332**	**0.6315**	**0.6132**	**0.6270**	0.7976	**0.7548**	**0.7703**

##### 3.2.2.2 CWFID dataset

**Fig. 7:**
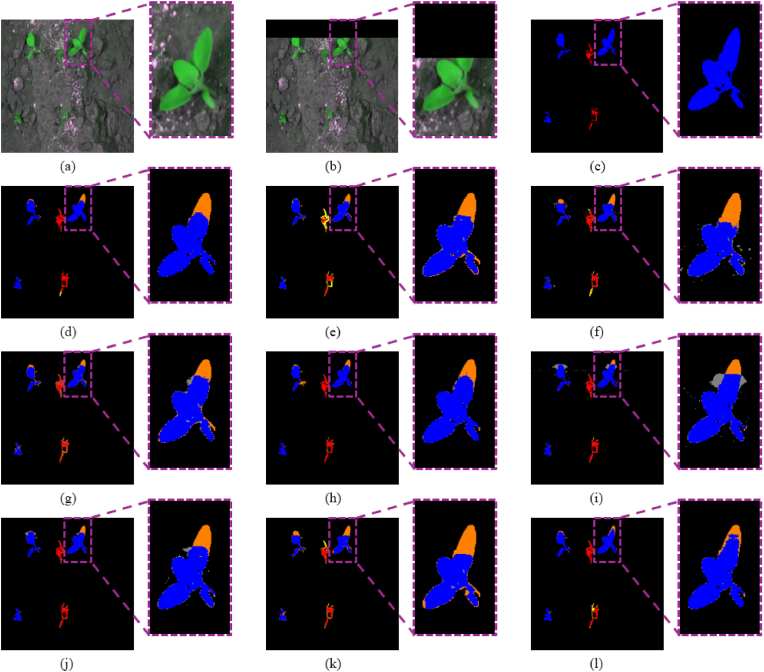
Comparison of semantic segmentation results between SOTA methods and KDOSS-Net on the CWFID dataset. Red, blue, and black indicate crops, weeds, and background, respectively. Yellow represents errors of crops being incorrectly identified as weeds or background whereas orange means errors of weeds being incorrectly detected as crops or background. Gray represents errors of the background being incorrectly identified as crops or weeds. (a) Original image; (b) limited FOV image; (c) ground truth label. Result images by (d) U-Net, (e) Deeplabv3+, (f) SegNet, (g) FCN-8s, (h) reduced U-Net, (i) modified U-Net, (j) SC-Net, (k) Segformer-B0, and (l) KDOSS-Net (proposed methods).

In this subsection, we compare the proposed method in KDOSS-Net with various existing crop and weed segmentation approaches on the CWFID dataset. Table 6 presents the quantitative comparison results, while Fig. 7 shows the qualitative comparison of semantic segmentation results between SOTA methods and KDOSS-Net. As shown in Table 6, the proposed method achieves the highest performance in *Accuracy*, *mIOU*, Weed *IOU*, *Precision*, and *F1 score*. Compared to U-Net, the second-best model in terms of *mIOU*, the proposed method demonstrates an improvement of 1.46 ​% in *mIOU*. Although the proposed method shows slightly lower performance in Crop *IOU* compared to U-Net, this can be attributed to the characteristics of the CWFID dataset, where the weed class is more prevalent. Due to the loss of information in the limited FOV, a significant portion of this loss occurs in the weed class. Compared to the proposed method, U-Net captures the crop class in the inside FOV better, resulting in higher Crop *IOU*, but does not capture the weed class in the outside FOV well, resulting in lower Weed *IOU* than the proposed method. Consequently, the proposed method achieves higher overall *mIOU* performance. Also, the proposed method shows lower performance in *Recall* compared to the modified U-Net. This is because the modified U-Net tends to predict a broader region as the positive class, thereby capturing more true positives and achieving higher *Recall*. However, this also increases the number of false positives by misclassifying background regions, resulting in lower *Precision* compared to the proposed method. As a result, the proposed method outperforms the modified U-Net in terms of *F1 score*. As shown in the zoomed-in regions of Fig. 7, despite the relatively simple shape of the leaves, other comparison models failed to accurately detect objects in the out-of-FOV regions, whereas the proposed model demonstrated the fewest errors in detecting these areas.

**Table 6:** Comparative accuracies of semantic segmentation on the CWFID dataset with SOTA segmentation models and the proposed KDOSS-Net.

Methods	*Accuracy*	mIOU	Crop IOU	Weed IOU	Recall	Precision	F1score
U-Net [14]	0.9786	0.6955	**0.5361**	0.7063	0.8079	0.8215	0.8130
Deeplabv3+ [16]	0.9730	0.6365	0.4359	0.6682	0.7519	0.7966	0.7720
SegNet [12]	0.9754	0.6732	0.4904	0.6898	0.8058	0.7982	0.8002
FCN-8s [9]	0.9685	0.6011	0.4254	0.6290	0.7273	0.7661	0.7448
Reduced U-Net [26]	0.9772	0.6767	0.5173	0.6988	0.7961	0.8097	0.8010
Modified U-Net [27]	0.9763	0.6689	0.4952	0.6931	**0.8154**	00.7827	0.7973
SC-Net [25]	0.9741	0.6426	0.4736	0.6834	0.7883	0.7605	0.7726
Segformer-B0 [19]	0.9674	0.5096	0.1502	0.5902	0.6377	0.6895	0.6612
KDOSS-Net (proposed)	**0.9800**	**0.7101**	0.5034	**0.7340**	0.8118	**0.8437**	**0.8259**

##### 3.2.2.3 BoniRob dataset

**Fig. 8:**
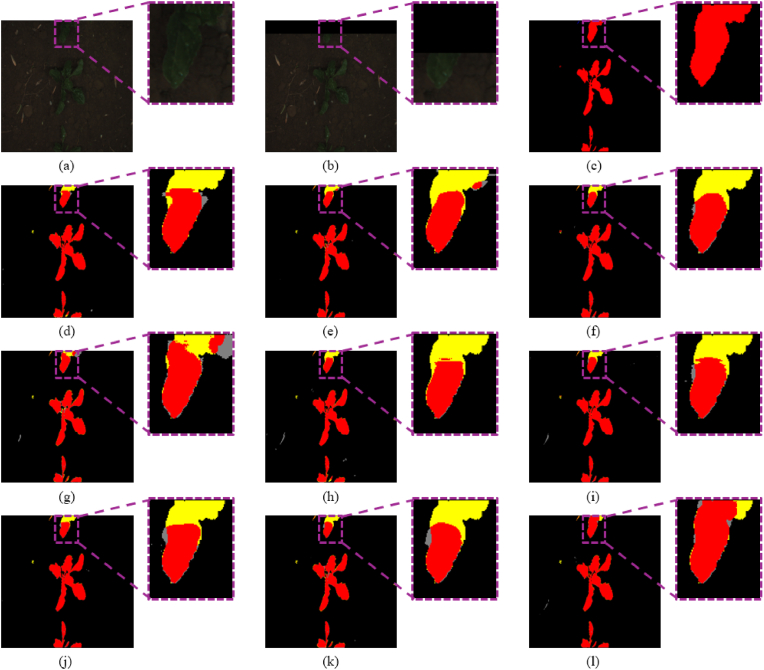
Comparison of semantic segmentation results between SOTA methods and KDOSS-Net on the BoniRob dataset. Red, blue, and black indicate crops, weeds, and background, respectively. Yellow represents errors of crops being incorrectly identified as weeds or background whereas orange means errors of weeds being incorrectly detected as crops or background. Gray represents errors of the background being incorrectly identified as crops or weeds. (a) Original image; (b) limited FOV image; (c) ground truth label. Result images by (d) U-Net, (e) Deeplabv3+, (f) SegNet, (g) FCN-8s, (h) reduced U-Net, (i) modified U-Net, (j) SC-Net, (k) Segformer-B0, and (l) KDOSS-Net (proposed methods).

In this subsection, the proposed KDOSS-Net is compared with existing crop and weed segmentation methods on the BoniRob dataset. The quantitative comparison results are presented in Table 7, while Fig. 8 shows the qualitative comparison of semantic segmentation results between the SOTA methods and KDOSS-Net. As shown in Table 7, the proposed KDOSS-Net achieved the highest performance in terms of *mIOU*, Crop *IOU*, Weed *IOU*, and *F1 score*. Compared to the second-best model in terms of *mIOU*, U-Net, KDOSS-Net outperformed it by 0.18 ​%. Furthermore, as shown in Table S11 (Supplementary Materials), KDOSS-Net achieves this performance with approximately half the number of parameters compared to U-Net. The proposed method showed lower *Accuracy* and *Precision* compared to U-Net. This is because *Accuracy*, as a metric reflecting overall pixel-wise correctness, is highly influenced by the background class, which constitutes most of the dataset. This suggests that U-Net may outperform the proposed method in distinguishing background regions more precisely. However, the proposed method achieved higher crop *IOU*, weed *IOU*, and *mIOU* compared to U-Net, indicating that while its ability to distinguish background may be inferior, it demonstrates superior performance in distinguishing object classes. In addition, the higher *Precision* of U-Net can be attributed to its training tendency to focus on accurate predictions by minimizing false positives. However, this often leads to a failure in detecting actual target classes, resulting in lower *Recall* compared to the proposed method. Consequently, the proposed method outperformed U-Net in terms of *F1 score*. In addition, the proposed method showed lower *Recall* compared to SegNet. This is because SegNet tends to predict a wider area as the positive class, capturing more true positives. However, this also leads to an increase in false positives due to misdetection of background regions, resulting in lower *Precision* compared to the proposed method. Ultimately, the proposed method outperformed SegNet in terms of *F1 score*. In addition, as shown in Fig. 8, the proposed method shows the most accurate results in the out-of-FOV regions, demonstrating its superior capability in predicting objects beyond the limited FOV.

**Table 7:** Comparative accuracies of semantic segmentation on the BoniRob dataset with SOTA segmentation models and the proposed KDOSS-Net.

Methods	*Accuracy*	mIOU	Crop IOU	Weed IOU	Recall	Precision	F1score
U-Net [14]	**0.9862**	0.7506	0.7897	0.5611	0.8102	**0.9059**	0.8523
Deeplabv3+ [16]	0.9838	0.7185	0.7753	0.4949	0.7978	0.8681	0.8288
SegNet [12]	0.9852	0.7436	0.7838	0.5629	**0.8294**	0.8728	0.8475
FCN-8s [9]	0.9804	0.6647	0.7478	0.3730	0.7464	0.8447	0.7884
Reduced U-Net [26]	0.9858	0.7431	0.7848	0.5443	0.8013	0.9050	0.8469
Modified U-Net [27]	0.9853	0.7415	0.7835	0.5419	0.8278	0.8716	0.8456
SC-Net [25]	0.9837	0.7212	0.7709	0.5074	0.8113	0.8592	0.8307
Segformer-B0 [19]	0.9804	0.6582	0.7356	0.3598	0.7327	0.8544	0.7828
KDOSS-Net (proposed)	0.9861	**0.7524**	**0.7905**	**0.5735**	0.8225	0.8940	**0.8540**

#### 3.2.3 Comparisons of KD by proposed and SOTA methods

In this subsection, the performance of SOTA KD methods is compared with the proposed KD approach in KDOSS-Net. Comparisons are conducted on three datasets: Rice seedling and weed, CWFID, and BoniRob. For logit-based KD [5,41], the method was measured using only logit-based KD as proposed in the respective papers. For feature-based KD [[42], [43], [44], [45], [46], [47], [48], [49]], the comparison was carried out using the same approach as in this study, which involves comparing the final encoder and decoder features as well as the logit. The performance for each dataset is presented in Table 8, Table 9, Table 10.

**Table 8:** Comparative accuracies of semantic segmentation on rice seedling and weed dataset with SOTA KD methods and the proposed KDOSS-Net.

Methods	*Accuracy*	mIOU	Crop IOU	Weed IOU	Recall	Precision	F1score
Teacher (standalone)	0.9330	0.6332	0.6133	0.6324	0.8013	0.7551	0.7712
Student (standalone)	0.9285	0.6071	0.5904	0.5983	0.7809	0.6786	0.7515
Logits [41]	0.9291	0.6189	0.6030	0.6090	**0.8063**	0.7312	0.7604
ST [5]	0.9266	0.6059	0.5918	0.5910	0.7967	0.7218	0.7499
FitNet [42]	0.9301	0.6222	0.6045	0.6172	0.8039	0.7375	0.7627
AT [43]	0.9294	0.6127	0.5975	0.6016	0.7885	0.7359	0.7556
FSP [44]	0.9297	0.6103	0.5949	0.5980	0.7791	0.7414	0.7539
SP [45]	0.9287	0.6070	0.5908	0.5998	0.7796	0.7358	0.7511
CWD [46]	0.9305	0.6187	0.6012	0.6126	0.7893	0.7474	0.7600
MLP [47]	0.9302	0.6218	0.6035	0.6193	0.8024	0.7383	0.7625
SRD [48]	0.9307	0.6163	0.6016	0.6014	0.7856	0.7436	0.7585
AttnFD [49]	0.9274	0.6067	0.5884	0.6016	0.7916	0.7275	0.7508
Proposed	**0.9332**	**0.6315**	**0.6132**	**0.6270**	0.7976	**0.7548**	**0.7703**

**Table 9:** Comparative accuracies of semantic segmentation on CWFID dataset with SOTA KD methods and the proposed KDOSS-Net.

Methods	*Accuracy*	mIOU	Crop IOU	Weed IOU	Recall	Precision	F1score
Teacher (standalone)	0.9811	0.7354	0.5543	0.7565	0.8288	0.8587	0.8422
Student (standalone)	0.9786	0.6824	0.4936	0.7173	0.7959	0.8177	0.8049
Logits [41]	0.9797	0.7081	**0.5363**	0.7328	0.8079	**0.8442**	0.8240
ST [5]	0.9791	0.7025	0.5251	0.7223	0.8093	0.8373	0.8212
FitNet [42]	0.9791	0.6954	0.5349	0.7082	0.7937	0.8413	0.8149
AT [43]	0.9762	0.6647	0.4813	0.6903	0.7808	0.8112	0.7941
FSP [44]	0.9768	0.6675	0.4652	0.6973	0.7792	0.8172	0.7952
SP [45]	0.9794	0.7060	0.5270	0.7309	0.8016	0.8497	0.8232
CWD [46]	0.9787	0.6926	0.5114	0.7183	0.7854	0.8470	0.8131
MLP [47]	0.9798	0.7095	0.5414	0.7302	**0.8173**	0.8357	0.8247
SRD [48]	0.9756	0.6764	0.4749	0.7026	0.8033	0.8064	0.8027
AttnFD [49]	0.9781	0.6930	0.4995	0.7222	0.7993	0.8324	0.8139
Proposed	**0.9800**	**0.7101**	0.5034	**0.7340**	0.8118	0.8437	**0.8259**

**Table 10:** Comparative accuracies of semantic segmentation on BoniRob dataset with SOTA KD methods and the proposed KDOSS-Net.

Methods	*Accuracy*	mIOU	Crop IOU	Weed IOU	Recall	Precision	F1score
Teacher (standalone)	0.9873	0.7708	0.8063	0.5934	0.8320	0.9086	0.8659
Student (standalone)	0.9806	0.7308	0.7741	0.5311	0.8014	0.8850	0.8380
Logits [41]	0.9859	0.7434	0.7873	0.5461	0.7962	0.9129	0.8468
ST [5]	0.9849	0.7306	0.7729	0.5313	0.7992	0.8878	0.8381
FitNet [42]	0.9857	0.7394	0.7860	0.5407	0.7907	**0.9138**	0.8440
AT [43]	0.9853	0.7351	0.7782	0.5362	0.8036	0.8897	0.8411
FSP [44]	0.9848	0.7268	0.7708	0.5255	0.7853	0.9014	0.8357
SP [45]	0.9854	0.7389	0.7807	0.5420	0.8051	0.8941	0.8440
CWD [46]	0.9857	0.7436	0.7831	0.5555	0.8049	0.9018	0.8474
MLP [47]	0.9855	0.7354	0.7823	0.5322	0.7887	0.9093	0.8409
SRD [48]	0.9762	0.7231	0.7732	0.5067	0.7837	0.8956	0.8327
AttnFD [49]	0.9852	0.7310	0.7781	0.5220	0.7887	0.9022	0.8378
Proposed	**0.9861**	**0.7524**	**0.7905**	**0.5735**	**0.8225**	0.8940	**0.8540**

##### 3.2.3.1 Rice seedling and weed dataset

In this subsection, the performance of various existing KD methods is compared with the proposed KD approach in KDOSS-Net using the Rice seedling and weed dataset. Table 8 presents the results of applying several existing KD methods and the proposed KD method, where *Accuracy*, *mIOU*, Crop *IOU*, Weed *IOU*, *Recall*, *Precision*, and *F1 score* are measured. The *Recall* value was higher for the Logits method, as it was trained to predict a wider area as the positive class, capturing more true positives. However, this led to an increase in false positives, causing a decrease in *Precision* compared to the proposed method. Ultimately, the *F1 score* demonstrated superior performance in the proposed method. Excluding *Recall*, the proposed method outperformed all other performance metrics. Additionally, the proposed method demonstrated superior performance compared to the standalone student model (without KD) and showed performance close to that of the standalone teacher model. In particular, the proposed method showed higher performance than the teacher model (standalone) in terms of Accuracy. Among the existing feature distillation methods, FitNet demonstrated the best performance in terms of *mIOU*, suggesting that minimizing the feature map differences between the teacher and student models is effective for this dataset. The proposed method in this paper outperformed FitNet, which minimizes the L2 distance between feature maps, by leveraging more channel information, thus enhancing performance.

##### 3.2.3.2 CWFID dataset

In this subsection, we compare the performance of various existing KD methods with the proposed method in KDOSS-Net on the CWFID dataset. Table 9 presents the segmentation performance results obtained by applying the various KD methods and the proposed KD method, following the same approach as in Section 3.2.3.1. The proposed method showed the best performance in terms of *Accuracy*, *mIOU*, Weed *IOU*, and *F1 score*. Additionally, the proposed method outperformed the student model (standalone) without KD across all performance metrics. In this dataset, the method that added an MLP to the student model to introduce non-linearity [47] showed the second-best performance after the proposed method in terms of *mIOU*. This suggests that distilling knowledge into the student model through non-linear activation functions and channel direction transformations via MLP is effective during KD. The MLP method outperformed the proposed method in terms of *Recall*, as the MLP approach encourages the model to predict a broader area as the positive class, capturing more true positives and thus achieving better *Recall*. However, this also led to an increase in false positives, resulting in a decrease in *Precision* compared to the proposed method. Ultimately, the proposed method outperformed the MLP method in terms of *F1 score*. Additionally, the Logits method demonstrated strong performance in terms of *mIOU*. This suggests that performing KD on the logits helps improve the consistency of the final classification results, which significantly contributes to enhancing the performance of the student model. Additionally, the Logits method showed better performance than the proposed method in terms of Crop *IOU* and *Precision*. This is because the weed class is more dominant in the dataset, and the proposed method tends to focus its learning on the weed class, which suffers from more information loss due to the FOV limitation. On the other hand, the Logits method focuses more on capturing the crop class, resulting in better performance in Crop *IOU*. However, for Weed *IOU*, the proposed method outperforms the Logits method. As a result, the proposed method achieves superior performance in *mIOU*. Additionally, the Logits method outperformed the proposed method in terms of *Precision*. This is because the Logits method was trained to minimize false positives, leading to a reduction in incorrect predictions and, consequently, higher *Precision*. However, this focus on minimizing false positives resulted in a decrease in *Recall* performance, as the model missed more true positives. Ultimately, the proposed method showed superior performance in terms of *F1 score*. The proposed method in this paper improves performance by utilizing the channel information from the teacher model more effectively. Unlike the MLP method, which adjusts the student model's channels to match the teacher model's channel count through nonlinear channel transformation, the proposed approach normalizes and expands the channels before distillation, thereby enhancing the model's overall performance.

##### 3.2.3.3 BoniRob dataset

In this subsection, we compare the proposed method in KDOSS-Net with several existing KD methods on the BoniRob dataset. Table 9 presents the segmentation performance results, applying various KD methods and the proposed KD approach, as measured in Sections 3.2, 3.2.3.1.3.2. The proposed method demonstrated the best performance in all metrics, except for *Precision*, and outperformed the student model (standalone) without KD across all performance indicators. In this dataset, CWD showed the second-best performance after the proposed method based on *mIOU*, and the Logits method also demonstrated good performance. This indicates that channel-wise distillation is effective when performing KD in the semantic segmentation method proposed in this study, and it also shows that the logit-based KD method achieves excellent performance. The proposed method in this paper achieved performance improvement by learning more comprehensive channel information from the teacher model compared to the CWD method, and by focusing more on channel information than the Logits method, further enhancing the performance. Additionally, the FitNet method outperformed the proposed method in *Precision*. This is because FitNet encourages the model to learn in a way that minimizes false positives, thus achieving better *Precision*. However, this approach resulted in more cases of failing to predict the correct class, leading to a lower *Recall* performance compared to the proposed method. Ultimately, the proposed method outperformed FitNet in terms of *F1 score*.

#### 3.2.4 Comparisons of processing time and computational cost

In this subsection, we compare the segmentation SOTA methods with the proposed method of this paper (SSWO-Net within KDOSS-Net) in terms of the number of model parameters, GPU memory requirements, floating point operations (FLOPs), inference time per image, and frames per second. First, measurements were performed on a desktop computer with the specifications described in subsection 2.3 (Table S11 in the Supplementary Materials). Subsequently, the inference time per image and frames per second were measured on the Jetson TX2 embedded system and mobile system (Samsung Galaxy S25+) (Table S12 in the Supplementary Materials). Jetson TX2 is an embedded system with NVIDIA Pascal™-family GPU (256 CUDA cores and less than 7.5 ​W of power consumption) with 8 ​GB shared memory between CPU and GPU. Fig. S7 (Supplementary Materials) illustrates the architecture of the Jetson TX2 embedded system. The measurements on the Jetson TX2 embedded system and mobile system were conducted to verify that the proposed model in this paper can operate on low computing power systems used in farming robots. In Table S11 (Supplementary Materials), the proposed SSWO-Net has the fourth smallest number of parameters among the compared SOTA models. Its GPU memory requirement is higher, but still lower than that of SC-Net. In terms of FLOPs, the proposed SSWO-Net has higher values than Reduced U-Net, Modified U-Net, and Segformer B0. However, its inference time and frames per second are lower than those of FCN, Reduced U-Net, and Segformer B0. Additionally, as shown in Table S12 (Supplementary Materials), when measured on the Jetson TX2, the proposed model outperformed U-Net, Deeplabv3+, SegNet, and SC-Net in terms of inference time and frames per second. Some of the lightweight models compared show better performance in terms of the number of model parameters, GPU memory requirement, FLOPs, inference time, and frames per second compared to the proposed model. However, as observed in Table 5, Table 6, Table 7, this comes at the cost of reduced segmentation performance. On the other hand, while the proposed model has slightly higher computational requirements compared to some of the lightweight models, it shows a clear advantage in segmentation accuracy, which is the primary goal of this study, as seen in Table 5, Table 6, Table 7 Additionally, as confirmed in Table S12, the proposed method can also operate on low computing power systems used in farming robots. Fig. S8 (Supplementary Materials) compares the segmentation accuracy, number of parameters, and FLOPs of the proposed method and SOTA methods using the rice seedling dataset for relative evaluation of computational cost. As shown in Fig. S8, the proposed model demonstrates high segmentation accuracy while also enhancing efficiency through model lightweighting.

## 4 Discussion

In this subsection, the semantic segmentation results are analyzed through gradient-weighted class activation mapping (Grad-CAM) [57], statistical analysis, and error case analysis. Section 4.1 discusses the analysis of segmentation results using Grad-CAM, Section 4.2 focuses on statistical analysis, and Section 4.3 addresses error cases. Additionally, Section 4.4 explores the application of a large language model (LLM) like LLaVA to assist non-experts in performing weed control.

### 4.1 Analysis with Grad-CAM

In this subsection, the analysis of segmentation results using Grad-CAM is discussed. In Grad-CAM, important features are displayed in red, while less important features are displayed in blue. The results using Grad-CAM are presented in Fig. S9 (Supplementary Materials). Fig. S9 (Supplementary Materials) shows the Grad-CAM images extracted from the last layer of the segmentation models that measured performance on the rice seedling and weed dataset. The proposed method (SSWO-Net within KDOSS-Net) extends the class activation to the out-of-view areas compared to the other comparison models, showing the most similar important features to the original training and testing case (Fig. S9 (d) (Supplementary Materials)). Moreover, it is observed that the proposed method not only focuses on the out-of-view information but also shows well-defined class activations for the in-view areas.

### 4.2 Analysis with statistical method

In this subsection, we discuss the analysis of results using statistical analysis. For the Rice seedling and weed dataset, a T-test [58] was performed and Cohen's d-value [59] was calculated to evaluate the statistical significance of the experimental results compared to semantic segmentation SOTA methods, as shown in Table 5. Cohen's d-value is categorized into small, medium, and large effect sizes based on the following thresholds: values close to 0.2 indicate a small effect size, values close to 0.5 represent a medium effect size, and values close to 0.8 indicate a large effect size. The mean and standard deviation of *mIOU* were calculated using the proposed method and the second-best method (SegNet). The results are presented in Fig. S10 (Supplementary Materials). The measured p-value was 0.042, indicating a statistically significant difference at the 95 ​% confidence level, and the Cohen's d-value was 3.21, showing a large effect size. These results confirm that the proposed method (SSWO-Net within KDOSS-Net) demonstrates significantly higher semantic segmentation accuracy compared to the second-best method.

### 4.3 Error cases of semantic segmentation by proposed method

**Fig. 9:**
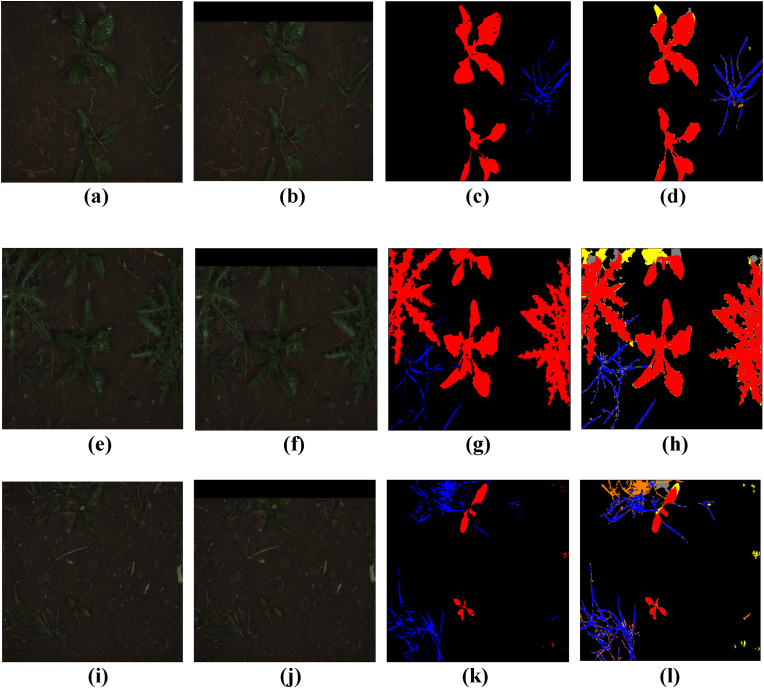
Examples of error cases by proposed method. Red, blue, and black indicate crops, weeds, and background, respectively. Yellow represents errors of crops being incorrectly identified as weeds or background whereas orange means errors of weeds being incorrectly detected as crops or background. Gray represents errors of the background being incorrectly identified as crops or weeds. (a), (e), (i) Original image; (b), (f), (j) limited FOV image; (c), (g), (k) ground truth. (d), (h), (l) Semantic segmentation results of limited FOV image by proposed method.

In this subsection, we discuss the error case analysis of the proposed SSWO-Net within KDOSS-Net. Examples of error cases are presented in Fig. 9. In Fig. 9 (d), segmentation errors occurred in images with limited FOV, where the object-background distinction was weak due to low illumination. This caused difficulties in predicting the orientation of the object, leading to some segmentation errors. In Fig. 9 (h) and (l), a relatively large number of errors occurred when many objects in crops or weeds were not visible due to the limited FOV. This indicates that the model's ability to predict the out-of-FOV regions is limited, especially for complex objects.

### 4.4 Comparative experiments according to occlusion methods

In this subsection, we additionally conducted experiments to compare the accuracies by random area occlusion. For experiments, we randomly selected the occlusion regions covering approximately 5–15 ​% of the area (whole image) for training and testing. The results are presented in Table S13 (Supplementary Materials). We observed that the segmentation accuracies by the occlusion of top 10 ​% of the area are similar to those by randomly occluding 5–15 ​% of the area, demonstrating that our proposed method is robust even under randomly occluded conditions.

### 4.5 Combining proposed method with LLaVA for AI-assisted weed management

**Fig. 10:**
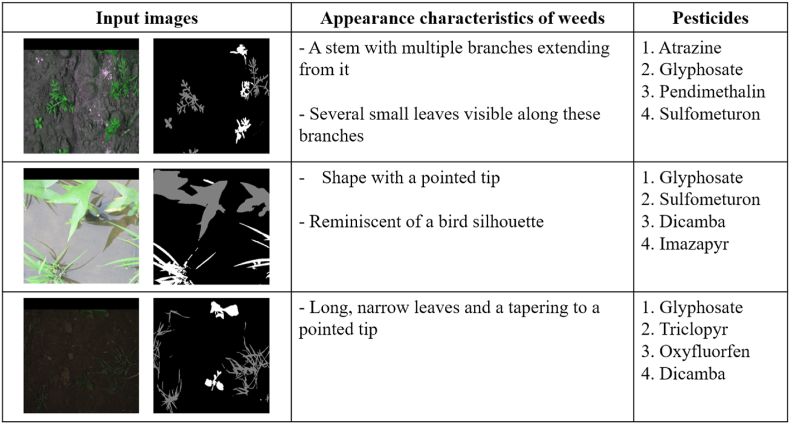
Pesticide recommendation system by LLaVA based on the input image and segmentation mask by proposed method. Appearance characteristics of weeds are obtained by LLAVA. Pesticides indicate the types and order of herbicides recommended by LLaVA based on the appearance characteristics of weeds.

In this subsection, we discuss how the segmentation results obtained by the proposed method are not only used for research purposes but are also integrated with a large language model (LLM) to make them easily accessible and useable by non-experts in agriculture. Recently, LLM-based AI systems have enabled non-experts to leverage professional-level analysis. Utilizing the segmentation results from the proposed method, we developed a pesticide recommendation system integrated with an LLM. That is, by inputting both the captured image and the segmentation result produced by the proposed method into the open-source LLaVA [60], the system can analyze the size, shape, and form of the weeds and recommend appropriate herbicides. An example of this functionality is shown in Fig. 10.

## 5 Conclusion

This study addresses the limitation of conventional segmentation models in capturing semantic information of crops and weeds located outside the visible field due to restricted FOV. To overcome this issue, we propose a semantic segmentation approach that incorporates information beyond the limited FOV. The proposed KDOSS-Net is a semantic segmentation framework designed to incorporate out-of-FOV information in crop and weed images. It consists of a teacher model, OPOSS-Net, which performs image outpainting followed by semantic segmentation, and a student model, SSWO-Net, which conducts segmentation without image restoration. The performance of the student model is enhanced through KD from the teacher. Ablation studies on the BoniRob dataset demonstrated that both the object prediction and image outpainting stages of OPOSS-Net effectively incorporated out-of-FOV information, thereby enhancing semantic segmentation performance. In addition, the proposed KD strategy was shown to improve the performance of the student model. The proposed KDOSS-Net was evaluated on three open crop and weed segmentation datasets—Rice seedling and weed, CWFID, and BoniRob—by comparing its performance with various SOTA semantic segmentation and KD methods. The results showed that KDOSS-Net achieved superior segmentation performance, with *mIOU* scores of 0.6315, 0.7101, and 0.7524 on the respective datasets, outperforming the other SOTA methods. In addition, the proposed method was demonstrated to be applicable to embedded and mobile systems with low computing power, considering deployment in farming robots. Moreover, Grad-CAM analysis confirmed that the proposed method effectively captures important features even in out-of-FOV regions. Statistical analysis further demonstrated that the proposed method achieved significantly higher semantic segmentation accuracy than the second-best method at a statistically meaningful level.

Additionally, a pesticide recommendation system integrated with the open-source LLM, LLaVA, was developed using the segmentation results from the proposed method. However, when the illumination is too low, there are limitations in predicting the directionality of crops and weeds based only on the information inside the FOV, resulting in segmentation errors. Additionally, when there are many objects that should be predicted outside the field of view, there are limitations in incorporating information from the external region, leading to a higher number of segmentation errors.

Therefore, in future research, we plan to investigate methods to predict a broader range of external FOV information to achieve better performance when many objects are located outside the field of view and are not visible. Additionally, we aim to further optimize the student model to make it lightweight while maintaining segmentation accuracy. Furthermore, we intend to expand the research by integrating the results of this study with various open-source LLMs, aiming to provide more comprehensive information and contribute to the development of AI-based smart farming systems.

## Author contributions

Conceptualization, S. H. Cheong; methodology, S. H. Cheong; validation, S. J. Lee, S. J. Im; software, J. Seo; supervision, K. R. Park; writing—original draft preparation, S. H. Cheong; writing—review and editing, K. R. Park. All authors have read and agreed to the published version of the manuscript.

## Declaration of competing interest

The authors declare that they have no known competing financial interests or personal relationships that could have appeared to influence the work reported in this paper.

## Data Availability

The data supporting the findings of this study can be accessed on GitHub [28].
