# Supplementary material for: KDOSS-net: Knowledge distillation-based outpainting and semantic segmentation network for crop and weed images
Source: Plant Phenomics. 2025 Aug 20;7(3):100098. doi: 10.1016/j.plaphe.2025.100098 (PMC12710004; doi:10.1016/j.plaphe.2025.100098)
Supplement: Multimedia component 1 [file mmc1.docx]

Supplementary Materials


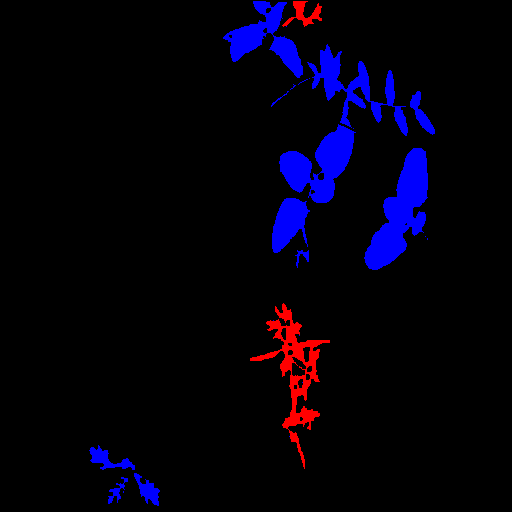


**(a)**


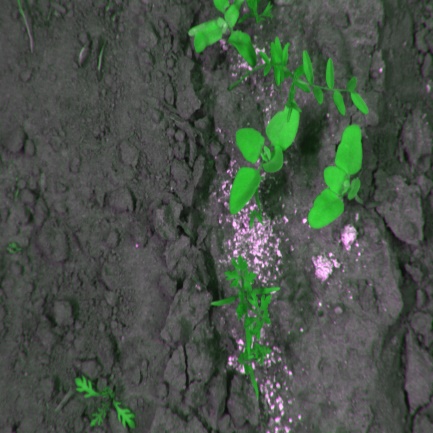

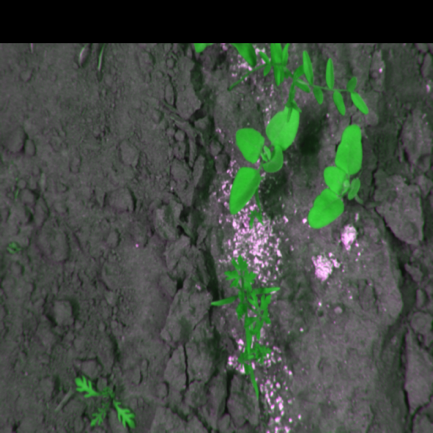


**(b) (c)**

**
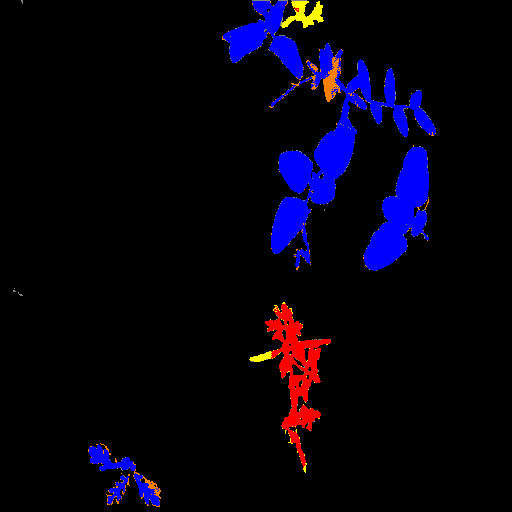

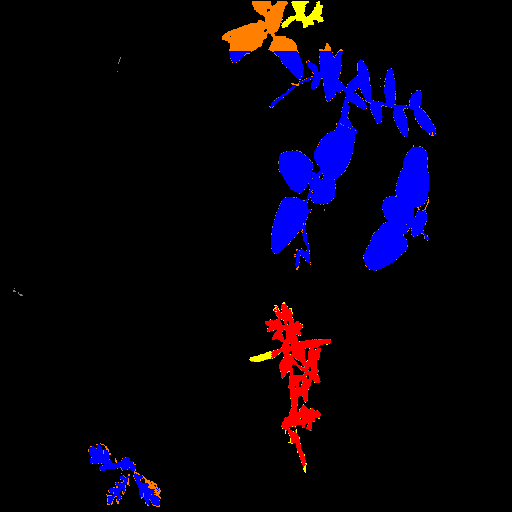
**

**(d) (e)**

Figure S1. Comparison of semantic segmentation results for the original and limited FOV images using U-Net [14]. Red, blue, and black indicate weeds, crops, and background, respectively. Yellow indicates error pixels where crops were incorrectly detected as weeds or background; orange indicates error pixels where weeds were incorrectly detected as crops or background; and gray represents error pixels where the background was incorrectly detected as crops or weeds. (a) Ground truth; (b) original image; (c) limited-FOV image; (d) semantic segmentation result of (b); (e) semantic segmentation result of (c).

**
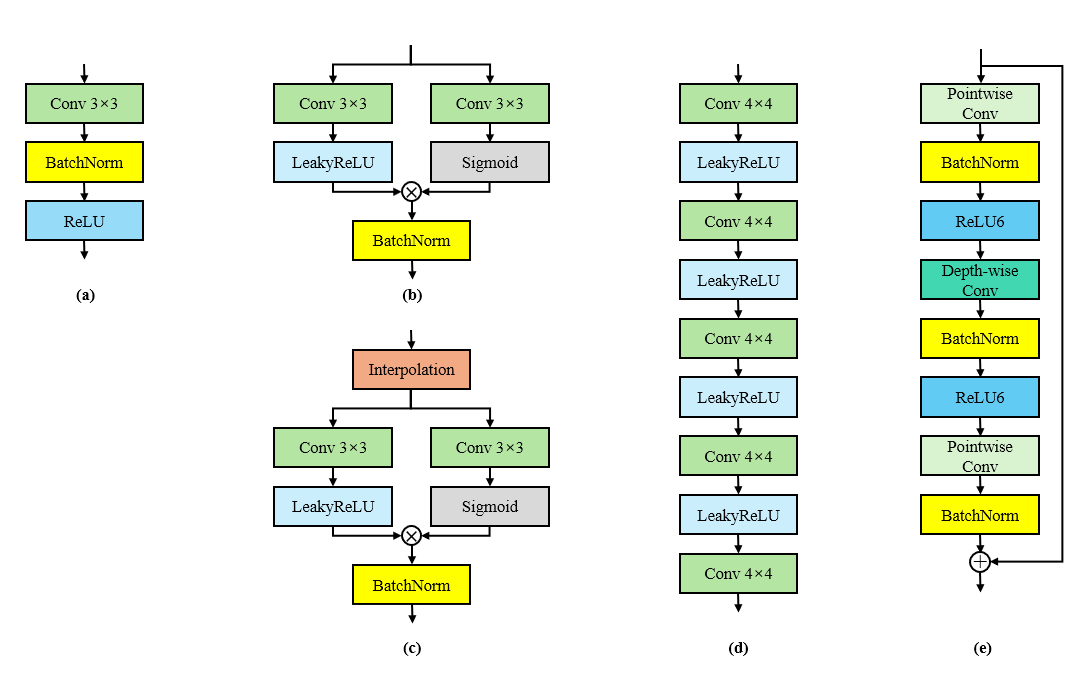
**

**Figure S2.** Detailed architecture of the (a) convolution block, (b) gated convolution block, (c) gated deconvolution block, (d) discriminator of the image outpainting network, and (e) inverted residual block.

| **Input Image** | **Ground-truth Label**  **(White: crop, Gray: weed)** |
| --- | --- |
| 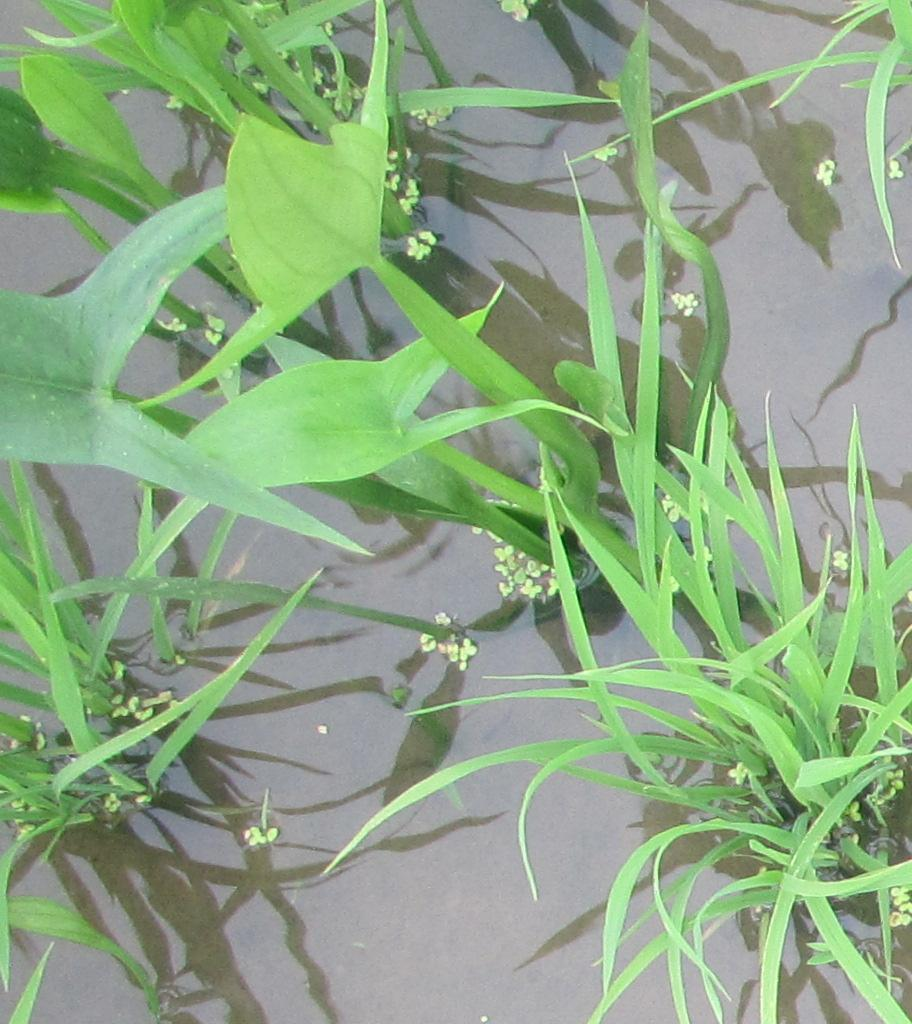 | 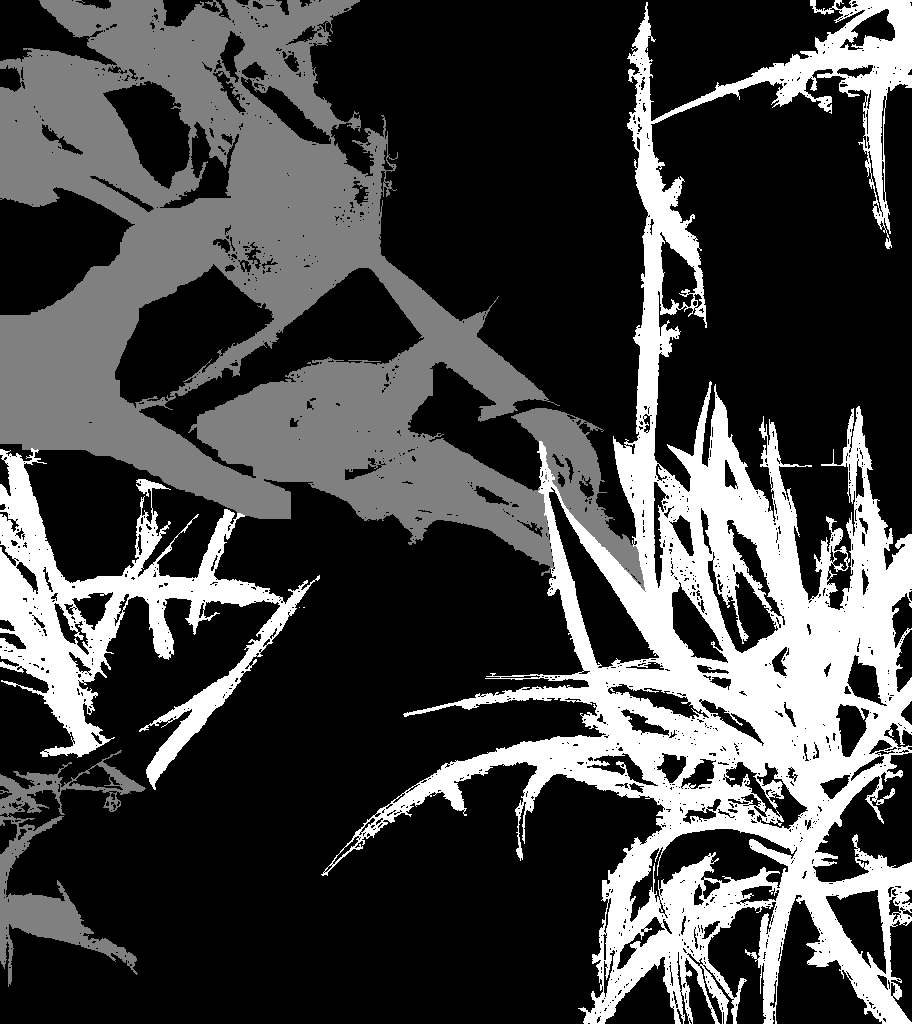 |
| **(a)** | |
| 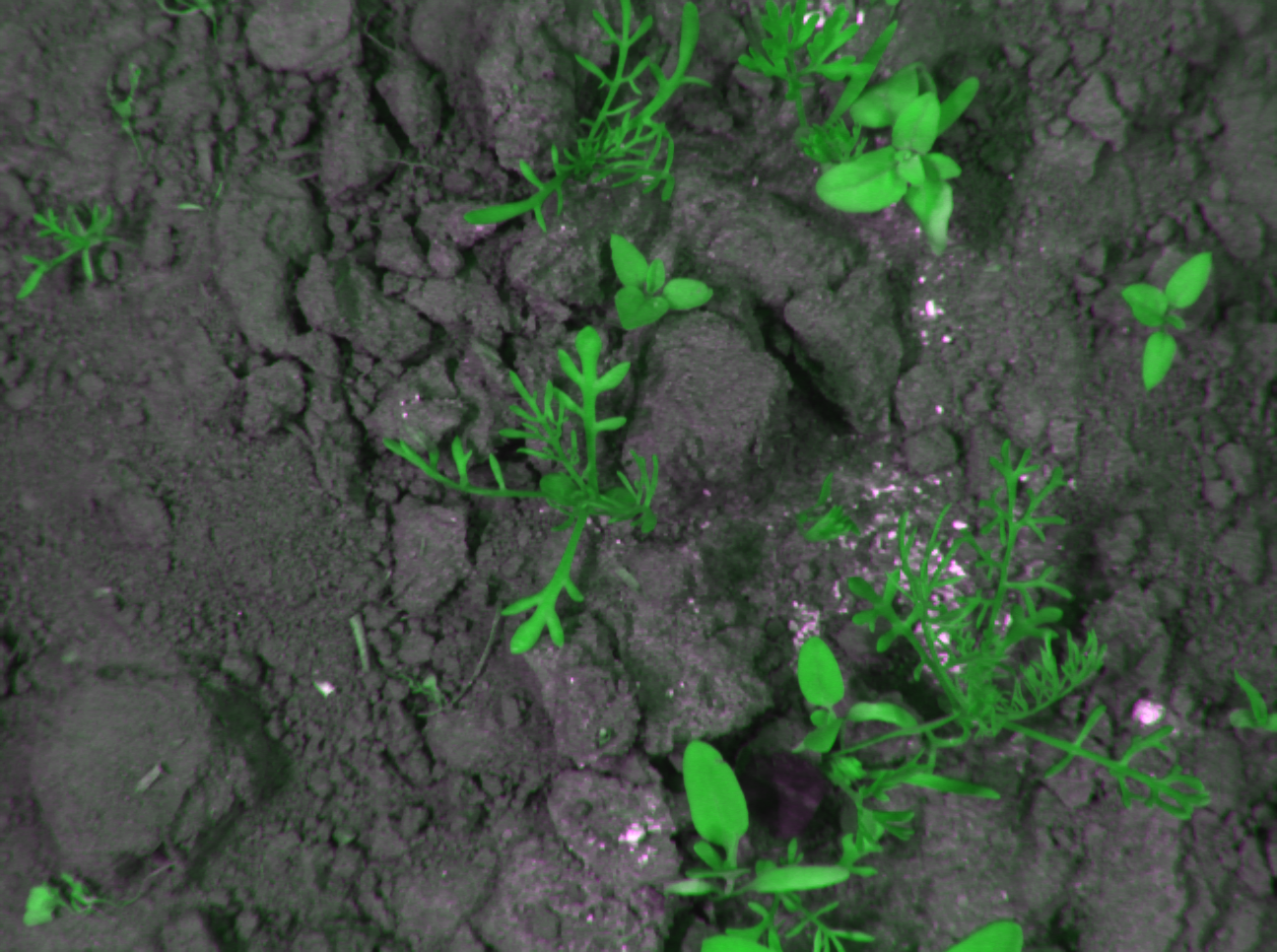 | 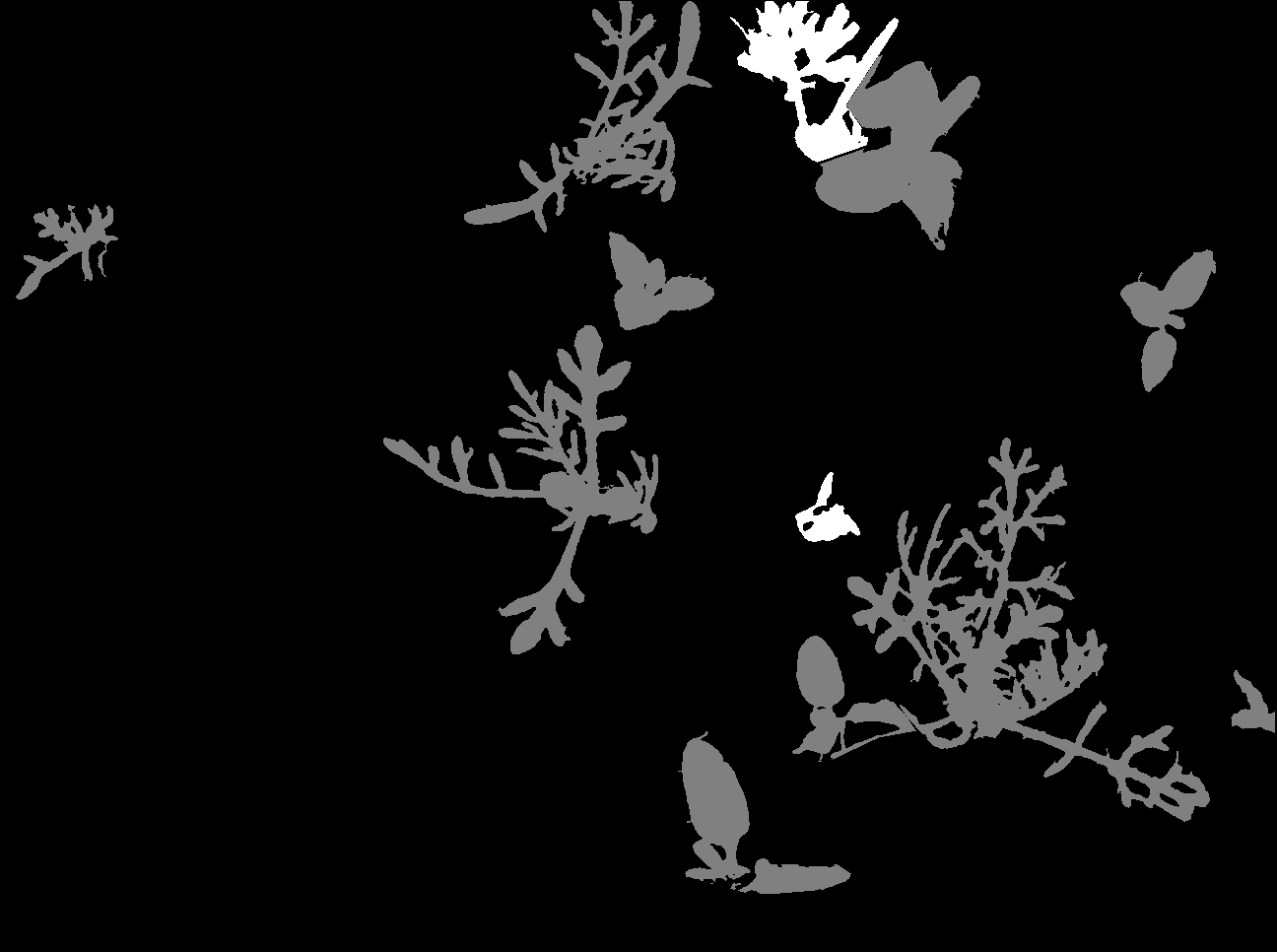 |
| **(b)** | |
| 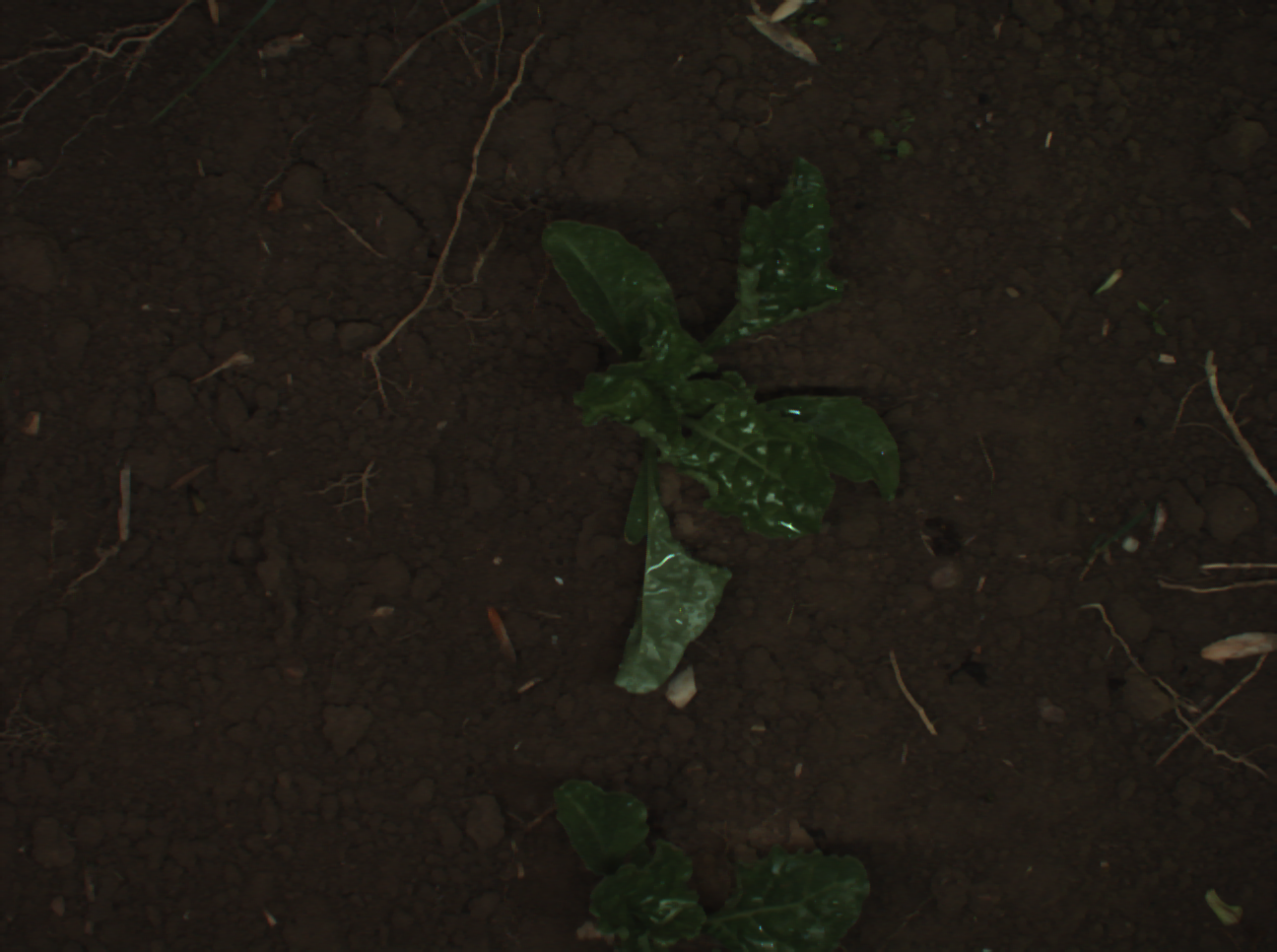 | 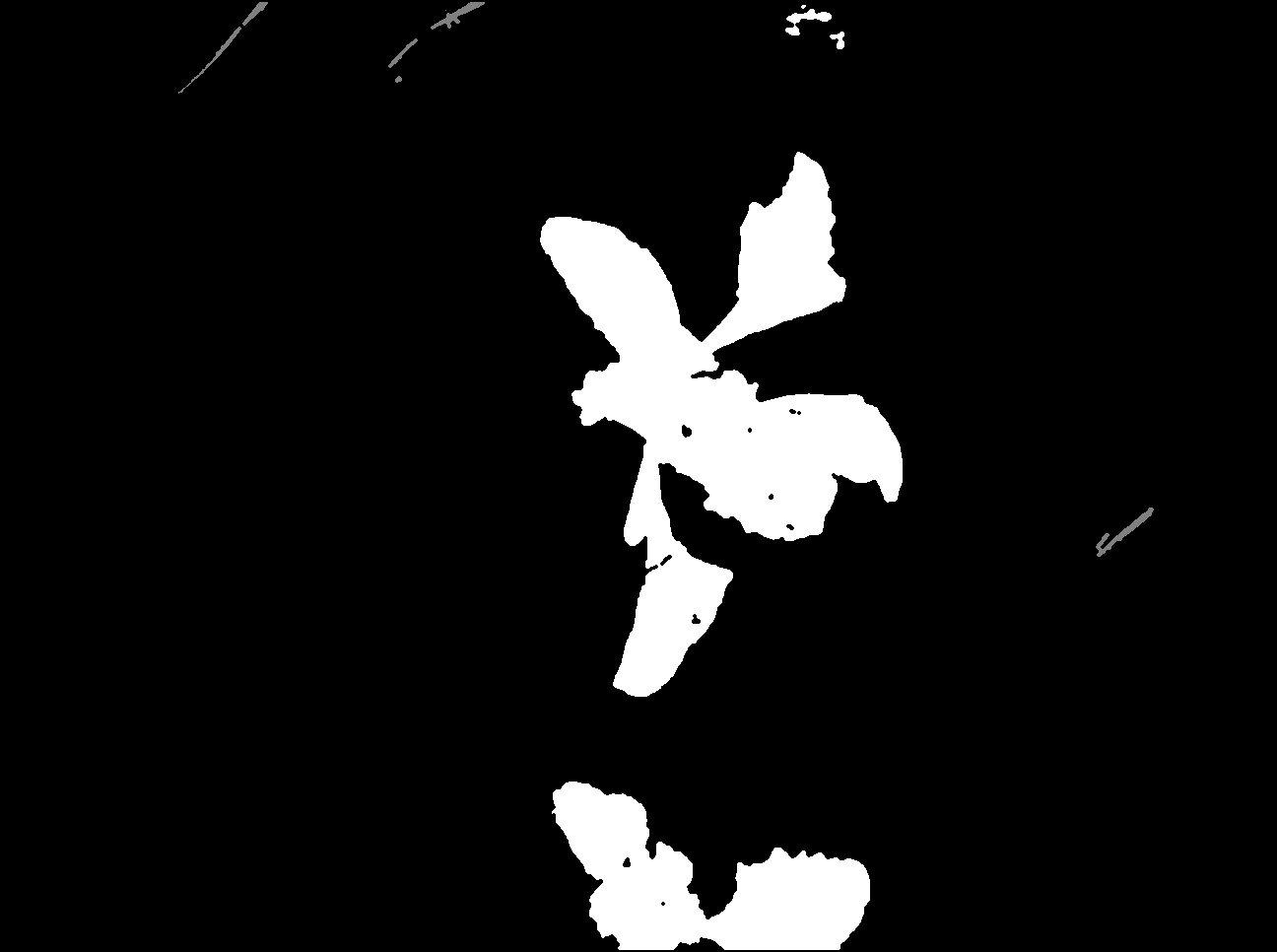 |
| **(c)** | |

Figure S3. Samples from crop and weed open datasets. (a) Rice seedling and weed dataset; (b) CWFID dataset; (c) BoniRob dataset.


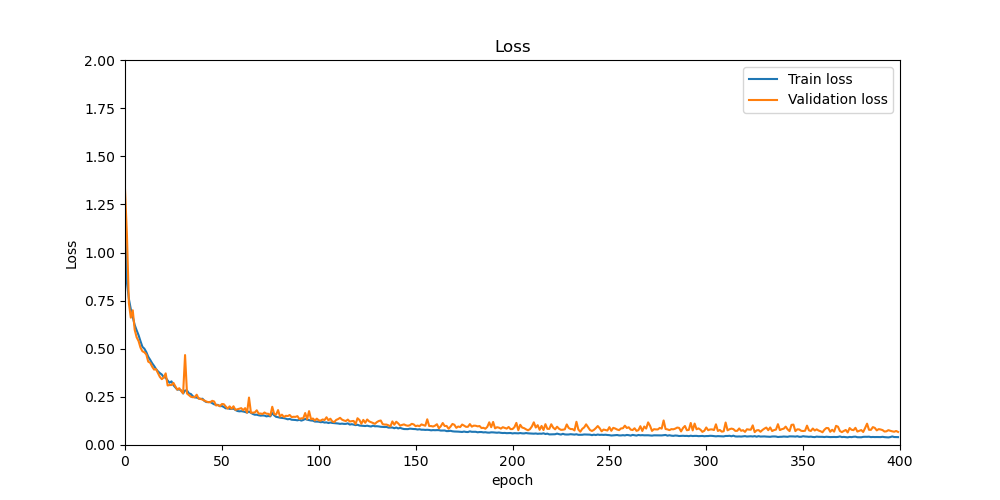


**Figure S4**. Training (blue line) and validation (orange line) loss graphs.


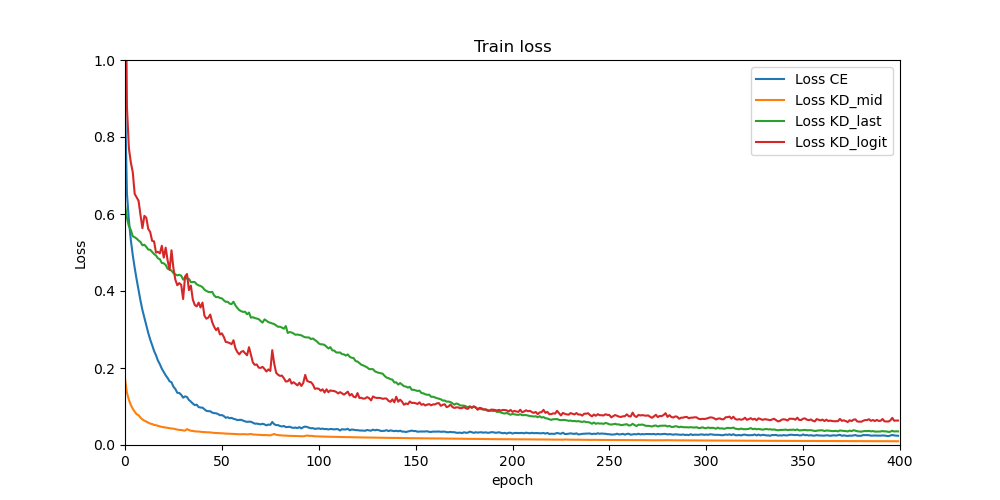


**Figure S5**. Detailed training loss graphs for each loss function. Blue indicates the cross-entropy loss, whereas orange, green, and red represent the mid, last, and logit components of the KD loss, respectively.


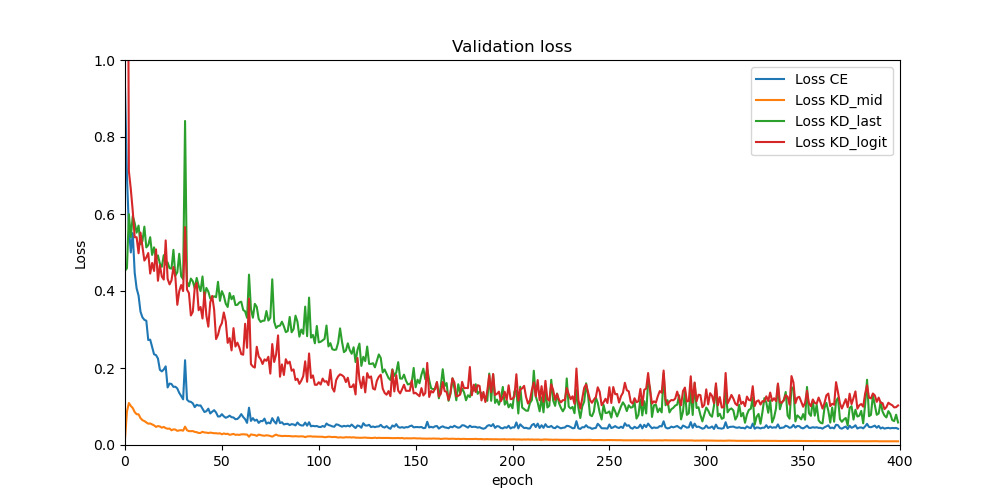


**Figure S6**. Detailed validation loss graphs for each loss function. Blue indicates the cross-entropy loss, whereas orange, green, and red represent the mid, last, and logit components of the KD loss, respectively.


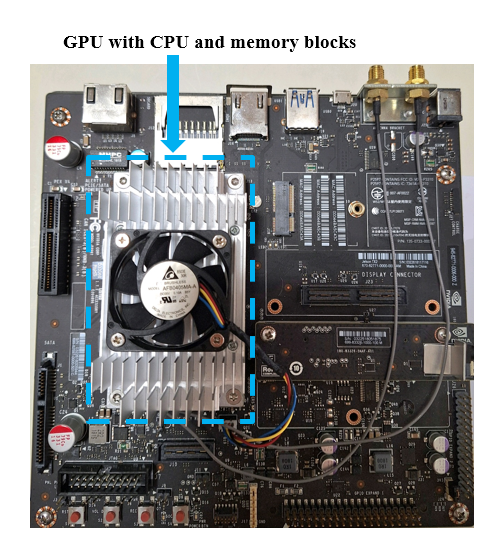


**Figure S7**. Jetson TX2 embedded system.


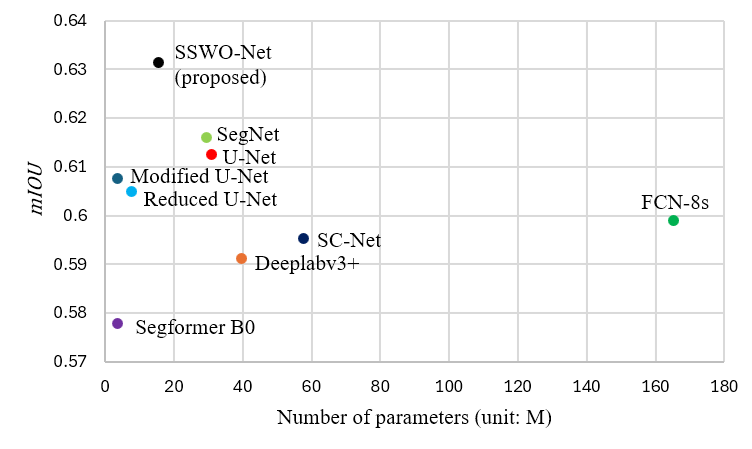


**(a)**

**
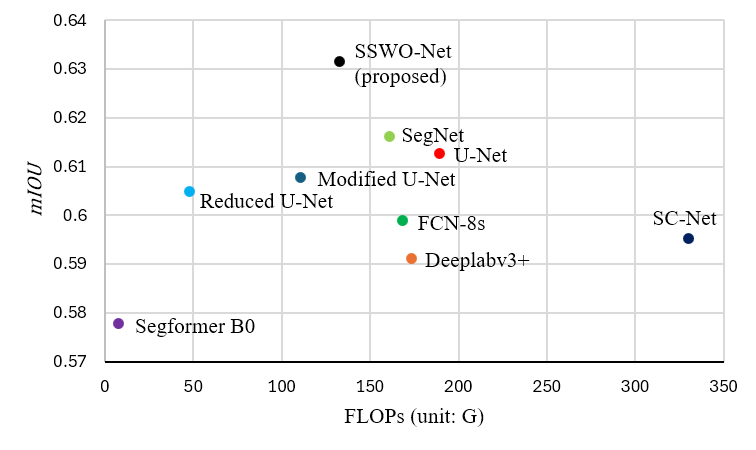
**

**(b)**

Figure S8. Comparison of the number of parameters with the unit of Mega, FLOPs with the unit of Giga, and semantic segmentation accuracy between SOTA models and the proposed method. (a) *mIOU* vs. number of parameters trade-off plot; (b) *mIOU* vs. FLOPs trade-off plot.


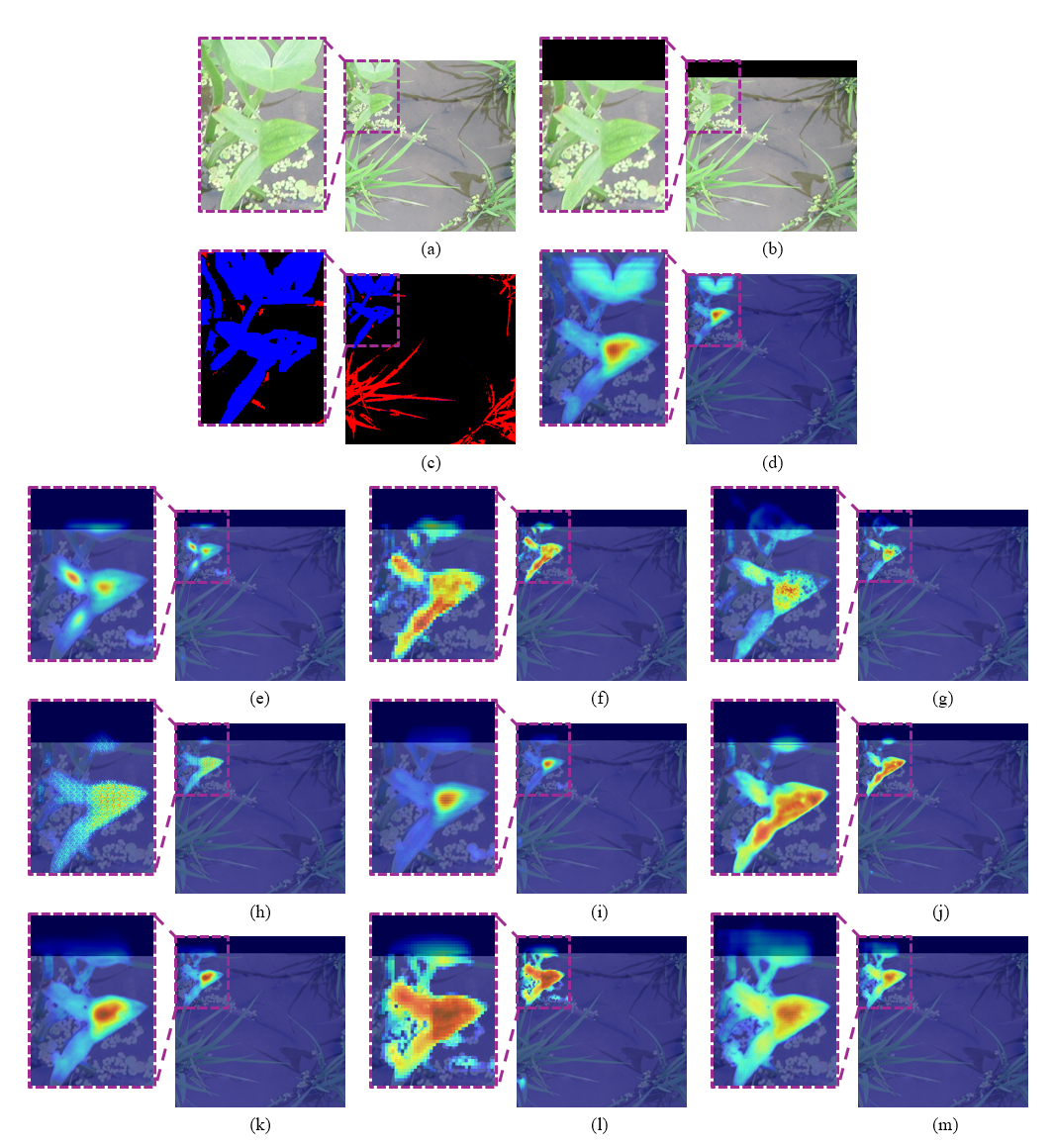


**Figure S9**. Grad-CAM comparisons between the proposed and SOTA methods on the rice seedling and weed dataset. (a) Original image; (b) limited field of view (FOV) image; (c) ground truth label; (d) U-Net trained with original images; (e) U-Net; (f) DeepLabv3+; (g) SegNet; (h) FCN-8s; (i) reduced U-Net; (j) modified U-Net; (k) SC-Net; (l) Segformer-B0; (m) KDOSS-Net (proposed).


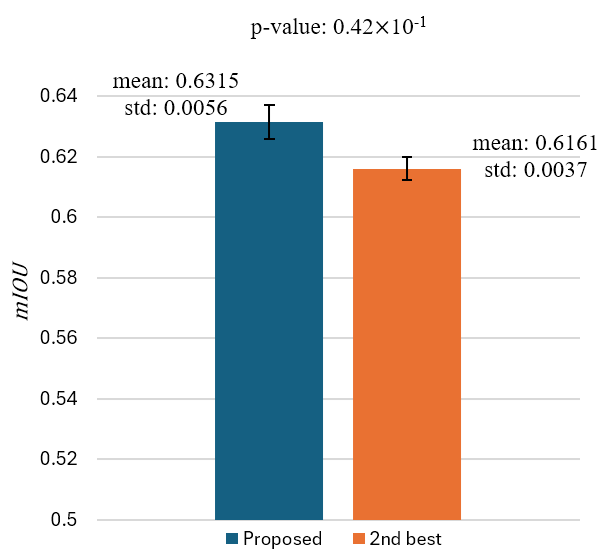


**Figure S10**. T-test results comparing semantic segmentation accuracy between the proposed method and the second-best method on the rice seedling and weed dataset.

| **Table S1.** Summary of comparisons between the proposed and previous works on crop and weed segmentation. | | | | |  |
| --- | --- | --- | --- | --- | --- |
| **Category** | | **Method** | **Advantage** | **Disadvantage** |  |
| Not considering the limited FOV | Handcrafted feature-based | Mahalanobis distance-based classification after RGB-to-HSI conversion [6] | Relatively robust under varying lighting conditions | Accuracy may decrease due to the exclusion of the green (G) channel |  |
|  |  | Background separation using NDVI-based threshold classification followed by random forest classification [7] | Less affected by lighting variations due to the use of NDVI images | If the NDVI image distribution is not bimodal, the binary image separating crop/weed from background may be incorrectly generated |  |
|  |  | Segmentation of crops and weeds from soil using ExG method and Otsu thresholding, followed by SVDD-based classification of weeds and crops [8] | Fast inference using only RGB channel values | Difficult to apply when the crop or weed color is not green, and thresholding may be inaccurate |  |
|  | Deep feature-based | Pixel-wise crop and weed classification using FCN [9] | Enables training using only RGB input images, offering high automation potential | High computational load may render real-time application difficult |  |
|  |  | Stem detection using FC-DenseNet with RGB and NIR image inputs [10] | Efficiently performs two tasks using a single encoder and two decoders | - Joint training of two tasks may interfere with each other  - Additional camera required for NIR image acquisition reduces overall efficiency |  |
|  |  | Report on the superiority of SegNet on the rice seedling dataset [12] | Demonstrated that SegNet is more effective than various existing models | Focused on performance reporting for a specific dataset, which may limit generalizability to other datasets |  |
|  |  | Use of various UAV-based images as input and application of U-Net [14] | Improved segmentation accuracy by utilizing various types of images | Requires an additional camera for NIR image acquisition, reducing overall efficiency |  |
|  |  | Report demonstrating the superiority of DeepLabv3+ across diverse UAS datasets [16] | Effectively segments objects at multiple scales | High computational and memory demands may render real-time application on lightweight devices difficult |  |
|  |  | Report demonstrating the superiority of SegFormer over various Transformer-based models [19] | SegFormer, a lightweight model, outperforms other Transformer-based models | Transformer-based models require large amounts of training data and may take longer for training and inference compared to conventional CNNs |  |
|  |  | Study applying Enet and SegNet using 14 input channels derived from transformed RGB images [22] | Achieved good performance across various imaging environments by utilizing 14 input channels | Generating the transformed 14-channel input takes time, and increasing the number of input channels raises the number of convolutional filters, thereby increasing computational cost |  |
|  |  | Proposal of CED-Net performing 4-stage segmentation [23] | Improved segmentation accuracy by using multiple stages to segment each class | Sequential training means that instability in one stage can affect the others |  |
|  |  | Proposal of MTS-CNN using two U-Nets [24] | Improved segmentation accuracy by clearly distinguishing object and background in the first stage | Using two stages may significantly increase computational load and memory requirements |  |
|  |  | Proposal of a new SC-Net based on U-Net [25] | Enhanced feature extraction capability and improved performance on slender targets, leading to better segmentation performance | Complex model structure makes training difficult, and model compression may be needed for real-time application |  |
|  |  | Proposal of a reduced U-Net with halved filter counts per convolution layer [26] | Reduced the number of convolution filters by half, decreasing total parameters to one-fourth | Reducing the number of convolution filters may degrade feature extraction performance |  |
|  |  | Proposal of a modified U-Net with simplified encoder and decoder [27] | Greatly reduced parameters by simplifying the encoding and decoding stages | Simplified structure may lead to reduced segmentation performance compared to the original U-Net |  |
| Considering the limited FOV |  | KDOSS-Net (method proposed in this study) | First to enable detection of out-of-FOV crops and weeds using KD-based outpainting | KD-based training requires a significant amount of time |  |
|  |  |  |  |  |  |

**Table S2.** Descriptions of the three open datasets used for training, validation, and testing.

| **Dataset** | **Subsets** | **Total** | **Training** | **Validation** | **Test** |
| --- | --- | --- | --- | --- | --- |
| Rice seedling and weed | Fold 1 | 224 | 101 | 11 | 112 |
|  | Fold 2 | 224 | 101 | 11 | 112 |
| CWFID | Fold 1 | 60 | 27 | 3 | 30 |
|  | Fold 2 | 60 | 27 | 3 | 30 |
| BoniRob | Fold 1 | 240 | 108 | 12 | 120 |
|  | Fold 2 | 240 | 108 | 12 | 120 |

**Table S3.** Hyperparameter settings used for training KDOSS-Net.

| **Network** | | **Initial learning rate** | $\boldsymbol{\beta}_{\boldsymbol{1}}$ | $\boldsymbol{\beta}_{\boldsymbol{2}}$ | **Batch size** | **Epochs** |
| --- | --- | --- | --- | --- | --- | --- |
| OPOSS-Net  (teacher) | Object prediction | 1 × 10^-4^ | 0.5 | 0.999 | 4 | 400 |
|  | Image outpainting |  | 0 | 0.9 | 2 | 200 |
|  | Semantic segmentation |  | 0.5 | 0.999 | 4 | 400 |
| SSWO-Net (student) | |  |  |  |  |  |

**Table S4.** Ablation study cases for the three subnetworks of OPOSS-Net.

| **Cases** | **Object prediction** | **Image outpainting** | **Semantic segmentation** |
| --- | --- | --- | --- |
| Case 1 |  |  | ✓ |
| Case 2 | ✓ |  | ✓ |
| Case 3 |  | ✓ | ✓ |
| Case 4 (proposed) | ✓ | ✓ | ✓ |

**Table S5.** Comparison of semantic segmentation accuracy with and without the proposed OPOSS-Net.

| **Methods** | | ***Accuracy*** | $\boldsymbol{mIOU}$ | **Crop**$\boldsymbol{IOU}$ | **Weed** $\boldsymbol{IOU}$ | $\boldsymbol{Recall}$ | $\boldsymbol{Precision}$ | $\boldsymbol{F1 score}$ |
| --- | --- | --- | --- | --- | --- | --- | --- | --- |
| Without  object prediction and image outpainting  in OPOSS-Net | Deeplabv3+ [17] | 0.9838 | 0.7185 | 0.7753 | 0.4949 | 0.7978 | 0.8681 | 0.8288 |
|  | SegNet [13] | 0.9847 | 0.7436 | 0.7838 | 0.5629 | 0.8294 | 0.8728 | 0.8475 |
|  | FCN [1] | 0.9807 | 0.6647 | 0.7478 | 0.3730 | 0.7464 | 0.8447 | 0.7884 |
|  | U-Net [3] | 0.9804 | 0.7506 | 0.7897 | 0.5611 | 0.8102 | 0.9059 | 0.8523 |
| With  object prediction and image outpainting  in OPOSS-Net | Deeplabv3+ [17] | 0.9851 | 0.7381 | 0.7907 | 0.5313 | 0.8095 | 0.8854 | 0.8432 |
|  | SegNet [13] | 0.9852 | 0.7503 | 0.7918 | 0.5716 | **0.8396** | 0.8699 | 0.8521 |
|  | FCN [1] | 0.9862 | 0.6693 | 0.7444 | 0.4040 | 0.7611 | 0.8340 | 0.7926 |
|  | U-Net [3] | **0.9873** | **0.7708** | **0.8063** | **0.5934** | 0.8320 | **0.9086** | **0.8659** |

**Table S6.** Comparisons of training time and segmentation accuracy of the teacher model when using end-to-end versus sequential training.

| **Methods** | ***Training time (hours)*** | ***Accuracy*** | $\boldsymbol{mIOU}$ | **Crop**$\boldsymbol{IOU}$ | **Weed** $\boldsymbol{IOU}$ | $\boldsymbol{Recall}$ | $\boldsymbol{Precision}$ | $\boldsymbol{F1 score}$ |
| --- | --- | --- | --- | --- | --- | --- | --- | --- |
| End-to-end training | 98.8 | 0.9841 | 0.7220 | 0.7630 | 0.5184 | 0.7958 | 0.8799 | 0.8319 |
| Sequential training | **71.2** | **0.9873** | **0.7708** | **0.8063** | **0.5934** | **0.8320** | **0.9086** | **0.8659** |

**Table S7.** Comparison of SSWO-Net performance with and without KD.

| **Methods** | ***Accuracy*** | $\boldsymbol{mIOU}$ | **Crop**$\boldsymbol{IOU}$ | **Weed** $\boldsymbol{IOU}$ | $\boldsymbol{Recall}$ | $\boldsymbol{Precision}$ | $\boldsymbol{F}\boldsymbol{1 score}$ |
| --- | --- | --- | --- | --- | --- | --- | --- |
| Teacher | 0.9873 | 0.7708 | 0.8063 | 0.5934 | 0.8320 | 0.9086 | 0.8659 |
| Student without KD | 0.9806 | 0.7308 | 0.7741 | 0.5311 | 0.8014 | 0.8850 | 0.8380 |
| Student with KD | **0.9861** | **0.7524** | **0.7905** | **0.5735** | **0.8225** | **0.8940** | **0.8540** |

**Table S8.** Comparison of SSWO-Net performance with and without channel expansion in KD.

| **Methods** | ***Accuracy*** | $\boldsymbol{mIOU}$ | **Crop**$\boldsymbol{IOU}$ | **Weed** $\boldsymbol{IOU}$ | $\boldsymbol{Recall}$ | $\boldsymbol{Precision}$ | $\boldsymbol{F}\boldsymbol{1 score}$ |
| --- | --- | --- | --- | --- | --- | --- | --- |
| Student without channel expansion | 0.9857 | 0.7436 | 0.7831 | 0.5555 | 0.8049 | **0.9018** | 0.8474 |
| Student with channel expansion | **0.9861** | **0.7524** | **0.7905** | **0.5735** | **0.8225** | 0.8940 | **0.8540** |

**Table S9.** Comparison of SSWO-Net performance with and without the MLP in KD.

| **Methods** | ***Accuracy*** | $\boldsymbol{mIOU}$ | **Crop**$\boldsymbol{IOU}$ | **Weed** $\boldsymbol{IOU}$ | $\boldsymbol{Recall}$ | $\boldsymbol{Precision}$ | $\boldsymbol{F}\boldsymbol{1 score}$ |
| --- | --- | --- | --- | --- | --- | --- | --- |
| Linear | 0.9860 | 0.7514 | 0.7886 | 0.5714 | 0.8200 | **0.8946** | 0.8529 |
| MLP | **0.9861** | **0.7524** | **0.7905** | **0.5735** | **0.8225** | 0.8940 | **0.8540** |

**Table S10.** Summary of cases based on the KD loss application location.

| **Cases** | **Logit** | **Mid** | **Last** |
| --- | --- | --- | --- |
| Case 1 |  |  |  |
| Case 2 | ✓ |  |  |
| Case 3 |  | ✓ |  |
| Case 4 |  |  | ✓ |
| Case 5 | ✓ | ✓ |  |
| Case 6 | ✓ |  | ✓ |
| Case 7 |  | ✓ | ✓ |
| Case 8 (proposed) | ✓ | ✓ | ✓ |

Table S11. Evaluation of computational cost for SSWO-Net and SOTA methods on a desktop computer.

| **Methods** | **Number of parameters**  **(Mega)** | **GPU memory requirement**  **(Giga-bytes)** | **FLOPs**  **(Giga)** | **Inference time**  **(ms)** | **Frames per second** |
| --- | --- | --- | --- | --- | --- |
| U-Net [14] | 31.04 | 2.33 | 189.42 | 13.71 | 72.90 |
| Deeplabv3+ [16] | 39.76 | 2.43 | 173.60 | 11.24 | 88.91 |
| SegNet [12] | 29.45 | 2.01 | 161.16 | 11.37 | 87.89 |
| FCN-8s [9] | 165.11 | 0.74 | 168.26 | 6.21 | 160.84 |
| Reduced U-Net [26] | 7.77 | 1.13 | 47.70 | **4.19** | **238.34** |
| Modified U-Net [27] | **3.44** | 1.20 | 110.32 | 11.00 | 90.83 |
| SC-Net [25] | 57.60 | 4.00 | 329.92 | 32.02 | 31.22 |
| Segformer-B0 [19] | 3.72 | **0.37** | **7.88** | 5.61 | 177.98 |
| SSWO-Net (proposed) | 15.40 | 2.85 | 132.68 | 10.76 | 92.90 |

Table S12. Comparisons of inference time per image and frames per second (FPS) between SSWO-Net and SOTA methods on the Jetson TX2 embedded system and a mobile system.

| **Method** | **Inference time (ms)** | | **FPS** | |
| --- | --- | --- | --- | --- |
|  | **Jetson embedded system** | **Mobile system** | **Jetson embedded system** | **Mobile**  **system** |
| U-Net [14] | 730.39 | 2,243 | 1.36 | 0.45 |
| Deeplabv3+ [16] | 966.00 | 6,634 | 1.03 | 0.15 |
| SegNet [12] | 594.59 | 1,462 | 1.68 | 0.68 |
| FCN-8s [9] | 331.05 | 777 | 3.02 | 1.29 |
| Reduced U-Net [26] | 249.67 | 695 | 4.00 | 1.44 |
| Modified U-Net [27] | 442.70 | 1,151 | 2.25 | 0.87 |
| SC-Net [25] | 1,957.28 | 7,821 | 0.51 | 0.13 |
| Segformer-B0 [19] | **172.94** | **674** | **5.78** | **1.48** |
| SSWO-Net (proposed) | 571.36 | 1,064 | 1.75 | 0.94 |

Table S13. Comparative experiments according to occlusion methods.

| **Methods** | ***Accuracy*** | $\boldsymbol{mIOU}$ | **Crop**$\boldsymbol{IOU}$ | **Weed** $\boldsymbol{IOU}$ | $\boldsymbol{Recall}$ | $\boldsymbol{Precision}$ | $\boldsymbol{F}\boldsymbol{1 score}$ |
| --- | --- | --- | --- | --- | --- | --- | --- |
| Random 5-15% | 0.9865 | 0.7595 | 0.7982 | 0.5739 | 0.8273 | 0.8973 | 0.8581 |
| Top 10% | 0.9861 | 0.7524 | 0.7905 | 0.5735 | 0.8225 | 0.8940 | 0.8540 |

Table S14. Comparative results according to different combinations of loss weights of Eq. (6) for the OPOSS-Net’s image outpainting generator.

| **Methods** | $\boldsymbol{\lambda}_{\boldsymbol{p}\boldsymbol{ixel}}$ | $\boldsymbol{\lambda}_{\boldsymbol{a}\boldsymbol{dv}}$ | $\boldsymbol{\lambda}_{\boldsymbol{I}\boldsymbol{DMRF}}$ | $\boldsymbol{\lambda}_{\boldsymbol{C}\boldsymbol{E}}$ | ***PSNR*** | ***SSIM*** | ***Accuracy*** | $\boldsymbol{mIOU}$ | **Crop**$\boldsymbol{IOU}$ | **Weed** $\boldsymbol{IOU}$ | $\boldsymbol{Recall}$ | $\boldsymbol{Precision}$ | $\boldsymbol{F}\boldsymbol{1 score}$ |
| --- | --- | --- | --- | --- | --- | --- | --- | --- | --- | --- | --- | --- | --- |
| Case 1 | 1 | 1 | 1 | 1 | 41.26 | 0.9702 | 0.9872 | 0.7677 | 0.8012 | 0.5974 | 0.8262 | **0.9113** | 0.8638 |
| Case 2 | 1 | 0.01 | 0.01 | 0.1 | 40.44 | **0.9815** | 0.9872 | 0.7696 | 0.8035 | 0.5933 | 0.8292 | 0.9096 | 0.8651 |
| Case 3 | 1 | 0.01 | 0.1 | 0.01 | **41.93** | 0.9772 | 0.9871 | 0.7673 | 0.7994 | **0.5975** | **0.8341** | 0.9005 | 0.8635 |
| Case 4 (proposed) | 1 | 0.1 | 0.01 | 0.01 | 39.97 | 0.9691 | **0.9873** | **0.7708** | **0.8063** | 0.5934 | 0.8320 | 0.9086 | **0.8659** |
